# Cell-state- and compartment-dependent PI3K/Akt redox signaling in chronic kidney disease: mechanisms and translational implications

**DOI:** 10.3389/fphar.2026.1951798

**Published:** 2026-09-02

**Authors:** Jianzong Xuan, Renhua Ding, Haiyin Zhang, Yiyuan Wang, Liangliang Wu, Yuan Liu, Jianrui Dong, Ran Hu

**Affiliations:** 1 Department of Nephrology, Tianchang Traditional Chinese Medicine Hospital, Tianchang, Anhui, China; 2 Department of Nephrology, The First Affiliated Hospital of Anhui University of Chinese Medicine, Hefei, China

**Keywords:** cell states, chronic kidney disease, oxidative stress, PI3K/Akt signaling, mitochondrial redox signaling, renal fibrosis, subcellular compartments, therapeutic targeting

## Abstract

Chronic kidney disease (CKD) progression results from coordinated pathological processes across diverse renal cell types and cannot be reduced to extracellular matrix accumulation alone. Tubular stress, disruption of the glomerular filtration barrier, microvascular rarefaction, immune activation, and interstitial remodeling collectively contribute to the progressive loss of renal structure and function. Although oxidative stress occurs throughout CKD progression, modern redox biology emphasizes that the effects of reactive oxygen species (ROS) depend on their source, concentration, chemical identity, duration, and subcellular localization. As a central hub in this redox regulatory network, the phosphoinositide 3-kinase/protein kinase B (PI3K/Akt) pathway integrates redox sensing with cell survival, metabolic reprogramming, inflammation, and fibrosis. In experimental systems, oxidative signals such as hydrogen peroxide (H_2_O_2_) can reversibly oxidize phosphatase and tensin homolog (PTEN) and other protein tyrosine phosphatases, thereby favoring localized phosphatidylinositol 3,4,5-trisphosphate (PIP3) accumulation and Akt activation. Activated Akt engages downstream effectors including nuclear factor erythroid 2-related factor 2 (Nrf2), forkhead box O (FOXO) transcription factors, mechanistic target of rapamycin (mTOR), glycogen synthase kinase 3 beta (GSK3β), nuclear factor kappa B (NF-κB), sterol regulatory element-binding proteins (SREBPs), autophagy-related pathways, and mitochondrial quality-control programs, thereby reshaping redox homeostasis and influencing injury and repair trajectories. PI3K/Akt signaling exhibits marked context dependence in CKD. The GSK3β/Nrf2 branch can enhance antioxidant defenses and limit inflammation, whereas sustained AKT serine/threonine kinase 1 (AKT1), mTOR complex 1 (mTORC1), mTOR complex 2 (mTORC2), or PI3K/Akt/mTOR/hexokinase II (HKII) signaling can promote maladaptive tubular repair, podocyte injury, and interstitial cell activation. PI3K/Akt therefore represents a key signaling node linking redox regulation, cell-state changes, and tissue remodeling. This review examines PI3K/Akt-mediated redox regulation across four dimensions: redox inputs, organellar microdomains, renal cell states, and clinical translation, while discussing therapeutic strategies tailored to specific cell states and spatial compartments.

## 1 Introduction

Chronic kidney disease (CKD) is characterized by progressive nephron loss and renal fibrosis, with tubular atrophy and interstitial fibrosis closely associated with declining kidney function (Humphreys, , 2018; Liu et al., 2018; Huang et al., 2023). This process involves dynamic, sustained interactions among tubular epithelial cells, podocytes, microvascular endothelial cells, immune cells, and interstitial cells, rather than being confined to interstitial cell activation or end-stage extracellular matrix (ECM) deposition (Liu et al., 2018; Gewin, 2018; Nagata, 2016; Perry and Okusa, 2016; Tang et al., 2019). When exposed to proteinuria, uremic toxins, high glucose, hypoxia, mechanical stress, and inflammatory cytokines, tubular cells exhibit mitochondrial dysfunction (Kang et al., 2015), dedifferentiation (Gewin, 2018), cell cycle arrest (Lovisa et al., 2015), secretory phenotype transition (Yang et al., 2010), and partial epithelial-mesenchymal transition (EMT) remodeling (Grande et al., 2015). Lovisa et al. (Lovisa et al., 2015) demonstrated through genetic lineage tracing and mouse renal fibrosis models that tubular epithelial EMT programs do not necessarily imply full transformation into fibroblasts; instead, they induce cell cycle arrest and parenchymal injury, thereby shaping a fibrotic microenvironment. Lake et al. (Lake et al., 2023) constructed a multimodal spatial atlas of the human kidney defining healthy and injured cell states along with their organizational neighborhoods, offering a reference for understanding cell state transitions in CKD. In 2024, Abedini et al. (Abedini et al., 2024) integrated human kidney single cell, multiomics, and spatial analyses to establish that the fibrotic microenvironment correlates intimately with CKD progression, shifting research focus from single molecular biomarkers toward cellular states and their spatial neighborhoods.

In this context, understanding the role of phosphoinositide 3 kinase/protein kinase B (PI3K/Akt) in CKD requires moving beyond single effect descriptions toward a context-dependent network perspective. Previous studies have highlighted the cytoprotective functions of PI3K/Akt and its nuclear factor erythroid 2 related factor 2(Nrf2), BCL2-associated agonist of cell death (BAD), and mitochondrial effector branches during oxidative stress and acute kidney injury. Under acute insults such as ischemia, hypoxia, or contrast media, PI3K/Akt activation promotes Nrf2-mediated antioxidant responses (Zhang et al., 2016; Tongqiang et al., 2016) and BAD phosphorylation (Liu C. et al., 2020), inhibiting caspase-dependent apoptosis (Liu C. et al., 2020) and pyroptosis driven by mitochondrial reactive oxygen species (ROS) (Li et al., 2024). Translocation of activated AKT serine/threonine kinase 1 (AKT1) to tubular mitochondria preserves oxidative phosphorylation and energy homeostasis, enhancing tubular cell tolerance against acute damage (Lin et al., 2021). Conversely, another line of investigation focuses on PI3K/Akt/Mechanistic target of rapamycin (mTOR) signaling in cell growth, metabolic reprogramming, and fibrosis. In podocytes, tightly controlled mTOR activity maintains structural integrity and filtration barrier function, whereas sustained Mechanistic target of rapamycin complex 1 (mTORC1) hyperactivation induces foot process effacement, proteinuria, and diabetic kidney disease progression (Inoki et al., 2011; Gödel et al., 2011). In fibroblasts and pericytes, PI3K/Akt/mTOR participates in transforming growth factor-beta (TGF-β) driven myofibroblast differentiation, metabolic reprogramming, and ECM production (Jiang et al., 2013; Li et al., 2015; Chen et al., 2023). In diabetic kidney disease, aberrant PI3K/Akt/mTOR signaling coincides with tubular autophagy impairment (Huang et al., 2016); in interstitial cells, CKD related stimuli such as TGF-β and trimethylamine N-oxide (TMAO) promote glycolytic reprogramming, proliferation, and ECM generation via Akt/mTOR pathways (Li et al., 2015; Chen et al., 2023; Kapetanaki et al., 2021).

This review organizes evidence along a central axis comprising redox inputs, subcellular microdomains, Akt effector branches, cell states, and disease stages to clarify the functional divergence of PI3K/Akt across distinct pathological settings. This framework resolves recurring superficial contradictions in CKD literature. For example, heparin-binding-site-deficient fibroblast growth factor 1(FGF1^ΔHBS)^ suppresses oxidative stress and inflammation to attenuate CKD through the PI3K/Akt/glycogen synthase kinase 3 beta (GSK3β)/Nrf2 axis (Wang et al., 2019), whereas genetic deletion of AKT serine/threonine kinase 1 gene *(Akt1)* reduces tubular apoptosis, EMT-like remodeling, and interstitial fibrosis during the Acute kidney injury (AKI)-to-CKD transition (Kim et al., 2021). Similarly, inhibiting aberrant mTOR signaling alleviates certain fibrotic or diabetic renal injuries, yet complete or excessive mTOR blockade compromises podocyte homeostasis and filtration barrier integrity (Inoki et al., 2011; Gödel et al., 2011). Furthermore, while Nrf2 activation demonstrated renoprotection in animal models and early clinical trials (Jiang et al., 2010; Zheng et al., 2011), bardoxolone methyl failed to reduce end-stage kidney disease (ESKD) or cardiovascular mortality in late stage diabetic CKD patients and increased heart failure safety risks (Pergola et al., 2011; de Zeeuw et al., 2013). These disparate findings underscore that PI3K/Akt related redox effects are governed by cell states, signal magnitude, duration, and disease stages, requiring evaluation within defined spatial and pathological contexts.

## 2 Molecular and organellar foundations of PI3K/Akt redox regulation

### 2.1 Redox sensitive phosphatases and spatiotemporal control of phosphatidylinositol 3,4,5-trisphosphate (PIP3)/Akt signaling

PI3K/Akt signaling is highly sensitive to cellular redox status, with phosphatase and tensin homolog (PTEN) serving as a representative upstream phosphatase regulating PIP3 levels and Akt activity. PTEN restricts Akt recruitment to the plasma membrane and its subsequent activation by dephosphorylating PIP3 to phosphatidylinositol 4,5-bisphosphate (PIP2) (Maehama and Dixon, 1998). Studies have shown that hydrogen peroxide (H_2_O_2_) generated after growth-factor stimulation can reversibly oxidize PTEN, transiently reducing its phosphatase activity and thereby promoting localized PIP3 accumulation and Akt activation (Maehama and Dixon, 1998; Leslie et al., 2003; Kwon et al., 2004). Other protein tyrosine phosphatases, such as protein tyrosine phosphatase 1 B (PTP1B), undergo similar redox regulation (Salmeen et al., 2003). These findings suggest that beyond mediating oxidative damage, ROS can modulate the activation threshold, duration, and spatial distribution of PI3K/Akt signaling by regulating localized phosphatase activity.

**FIGURE 1:**
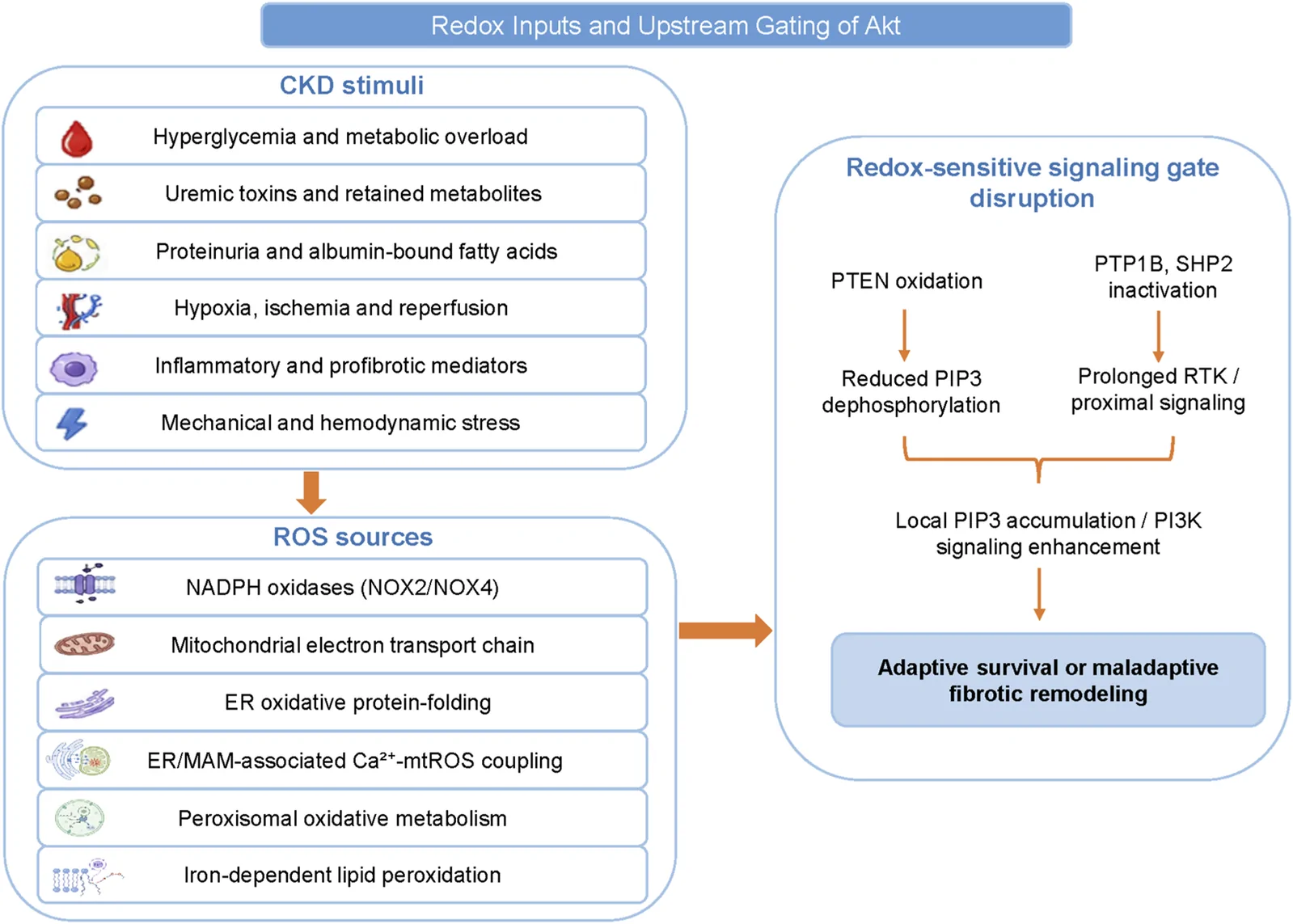
Redox inputs and upstream regulation of PI3K/Akt signaling in CKD. CKD-related stimuli, including hyperglycemia, uremic toxins, protein overload, hypoxia-ischemia, inflammatory mediators, and mechanical stress, increase ROS production from NADPH oxidases, mitochondria, the ER, mitochondria-associated membranes, peroxisomes, and lipid peroxidation. Local ROS can reversibly oxidize PTEN and inhibit other redox-sensitive phosphatases, thereby enhancing proximal receptor signaling, promoting local PIP3 accumulation, and facilitating Akt recruitment and activation. The resulting PI3K/Akt output varies with signal intensity, duration, subcellular localization, cell type, and disease stage, leading to either adaptive survival or maladaptive fibrotic remodeling.

In CKD environments, stimuli including high glucose, protein overload, hypoxia, uremic toxins, and inflammatory cytokines promote ROS production via nicotinamide adenine dinucleotide phosphate (NADPH) oxidases or mitochondria in tubular epithelial cells, podocytes, and vascular endothelial cells (Cao et al., 2011; Ishola et al., 2006; Eid et al., 2013; Casalena et al., 2020). However, direct evidence of reversible PTEN oxidation derives primarily from growth factor stimulation and non-renal cell models, lacking systematic validation regarding its oxidation state, subcellular localization, and actual function in diverse renal cell types during CKD. Thus, PTEN oxidation is best regarded as a foundational mechanistic model linking ROS to localized PI3K/Akt signaling rather than as a universally established mechanism in CKD (Figure 1). How ROS from distinct sources and locations further impact mitochondria, endoplasmic reticulum (ER), lysosomes, and lipid metabolism related organelles will be discussed below alongside organellar redox microdomains.

### 2.2 Organellar redox microdomains and cell fate

Intracellular ROS are not uniformly distributed; instead, they form redox microdomains with distinct sources and functions across different organelles and their contact sites. Mitochondria, ER, lysosomes, peroxisomes, and lipid droplets participate in energy metabolism, protein folding, calcium homeostasis, quality control, and lipid metabolism, respectively, exchanging substances and signals through organelle contact sites (Zhao et al., 2021; Bhatia et al., 2020; Marchi et al., 2014; Zou et al., 2020; Olzmann and Carvalho, 2019). Subcellularly localized PI3K/Akt signaling selectively influences mitochondrial metabolism, ER mitochondria calcium transfer, lysosomal mTORC1, autophagy, and lipid synthesis. Consequently, tissue or whole-cell level alterations in phosphorylated Akt (p-Akt) fail to capture its distinct functional outputs in CKD.

#### 2.2.1 Mitochondrial ROS and mitochondrial quality control

Mitochondria serve as vital hubs for energy metabolism and redox regulation in renal cells. Impaired electron transport chain function reduces oxidative phosphorylation and adenosine triphosphate (ATP) generation while increasing mitochondrial ROS production; damaged mitochondria can also release damage-associated molecular patterns (DAMPs) such as mitochondrial DNA (mtDNA), bridging metabolic dysfunction and inflammatory responses (Zhao et al., 2021; Bhatia et al., 2020). This process is critical for proximal tubular cells with high energy demands and contributes to state transitions in other cell types, though its relative contribution varies by cell type.

Once mtDNA or other cell-derived double-stranded DNA (dsDNA) enters the cytosol, it can be sensed by cyclic GMP-AMP synthase (cGAS), which signals through stimulator of interferon genes (STING). Activated cGAS generates cyclic GMP-AMP (cGAMP), which binds STING and activates the TANK-binding kinase 1 (TBK1)-interferon regulatory factor 3 (IRF3) and nuclear factor kappa B (NF-κB) branches, thereby inducing type I interferon and inflammatory gene programs. In experimental obstructive nephropathy, global cGAS deficiency, global or myeloid-specific STING deficiency, and pharmacological cGAS inhibition reduced macrophage proinflammatory activation, myofibroblast formation, collagen deposition, and renal fibrosis; damaged tubular epithelial cell-derived dsDNA was also shown to activate cGAS-STING signaling in macrophages (Jiao et al., 2025). These findings identify cytosolic DNA sensing as an additional link between organellar damage and renal inflammation, operating alongside inflammasome activation during chronic kidney disease (CKD) progression.

Akt and mitochondrial function are linked bidirectionally. Akt supports cell survival and energy metabolism through BAD, forkhead box O (FOXO), GSK3β, metabolic enzymes, and mitochondrially localized AKT1 (Lin et al., 2021). Conversely, sustained Akt/mTOR activity may increase anabolic demand and, when accompanied by impaired autophagy, reduce the clearance of damaged mitochondria and perpetuate mitochondrial ROS production.

Investigations in CKD also indicate that mitochondrial function depends on coordination among metabolic inputs, respiratory chain integrity, and quality control. Kayhan et al. (Kayhan et al., 2023) demonstrated that proximal tubule specific transforming growth factor-beta receptor type 2 (*Tgfbr2*) deletion impaired mitochondrial complex I expression and quality control, accompanied by reduced oxidative phosphorylation, glycolytic reprogramming, and aggravated inflammation; decreased TGF-β receptor expression and aberrant mitochondrial metabolic programs were also observed in human CKD proximal tubules. Although that study did not directly examine PI3K/Akt mechanisms, it underscores that evaluating mitochondrial homeostasis requires assessing respiratory chain integrity, quality control, and metabolic coupling rather than relying on isolated expression or biogenesis markers. Notably, the maintenance of mitochondrial and metabolic homeostasis by endogenous proximal tubular TGF-β receptor signaling does not contradict the pro-fibrotic effects of TGF-β on fibroblasts and pericytes during chronic injury, reflecting cell type and context-dependent TGF-β actions (Li et al., 2015; Chen et al., 2023; Kayhan et al., 2023).

#### 2.2.2 Endoplasmic reticulum/mitochondria-associated ER membranes (MAMs), calcium homeostasis, and protein-folding stress

The ER manages the folding of secretory and membrane proteins, lipid synthesis, and intracellular calcium storage (Schwarz and Blower, 2016). Oxidative stress, nutrient overload, and aberrant protein accumulation disrupt ER homeostasis, activating the unfolded protein response (UPR) (Walter and Ron, 2011). Transient UPR activation decreases protein synthesis, enhances chaperone expression, and restores folding capacity; persistent stress can trigger inflammation, metabolic dysfunction, and cell death (Walter and Ron, 2011; Hetz and Papa, 2018).

Protein folding within the ER is inherently a redox process. In the classical ER oxidoreductin 1 (ERO1) - protein disulfide isomerase (PDI) oxidative folding pathway, oxidized PDI catalyzes disulfide bond formation in nascent polypeptides and becomes reduced; ERO1 then re-oxidizes PDI and transfers electrons to molecular oxygen, generating H_2_O_2_ as a byproduct (Gross et al., 2006; Sevier et al., 2007). While supporting disulfide bond formation in secretory proteins, this reaction imposes localized oxidative pressure within the ER lumen. Peroxiredoxin 4 (PRDX4) utilizes H_2_O_2_ to re-oxidize PDI, coupling peroxide clearance with disulfide bond formation to limit excessive H_2_O_2_ accumulation (Zito et al., 2010). Thus, ER ROS contribute to controlled oxidative protein folding but can exacerbate proteostatic disruption when generation and clearance become imbalanced.

The ER and mitochondria form structural and functional connections via mitochondria-associated ER membranes (MAMs). MAMs facilitate Ca^2+^, phospholipid, and redox signal transfer, coupling ER proteostasis with mitochondrial metabolism, ROS generation, and cell death (Marchi et al., 2014). In HeLa cells and inducible rapamycin-insensitive companion of mTOR (*Rictor*) knockout mouse embryonic fibroblasts, mechanistic target of rapamycin complex 2 (mTORC2)/Akt signaling localizes to MAMs and restricts excessive ER Ca^2+^ release through Akt-dependent inositol 1,4,5-trisphosphate receptor type 3 (IP3R3) phosphorylation. Disruption of this signaling enhances mitochondrial Ca^2+^ uptake, induces abnormal elevations in mitochondrial membrane potential and ATP levels, compromises MAM integrity, and sensitizes cells to apoptotic stimuli (Betz et al., 2013). This mechanism provides a direct basis for PI3K/Akt involvement in ER mitochondria contact site regulation, though its generalizability across renal cell types in CKD requires cell-specific validation. ER stress in CKD should therefore not be viewed as isolated proteostatic dysfunction independent of mitochondria. MAM quantity, contact distance, Ca^2+^ transfer intensity, and localized mTORC2/Akt activity can all alter mitochondrial metabolism and ROS output.

#### 2.2.3 Autophagy and mitophagy

Autophagy degrades aberrant proteins, protein aggregates, and damaged organelles via lysosomes, recycling metabolic substrates such as amino acids and fatty acids (Mizushima and Komatsu, 2011). Lysosomes also serve as a spatial platform where mTORC1 integrates nutrient and growth factor signals (Mizushima and Komatsu, 2011; Sancak et al., 2008; Sancak et al., 2010). Amino acid sensing promotes mTORC1 recruitment to the lysosomal membrane via the Rag GTPase-Ragulator complex. Following growth factor activation, Akt phosphorylates and inhibits tuberous sclerosis complex 2 (TSC2), relieving TSC inhibition on ras homolog enriched in brain (Rheb) and enabling Rheb-GTP to activate mTORC1 on the lysosomal membrane (Inoki et al., 2002; Menon et al., 2014). Activated mTORC1 directly phosphorylates UNC-51 like autophagy activating kinase 1 (ULK1) to suppress autophagy initiation, whereas AMP-activated protein kinase (AMPK) activation during energy depletion relieves this suppression to promote ULK1 dependent autophagy (Kim et al., 2011). Thus, PI3K/Akt regulation of autophagy and anabolism exhibits clear lysosomal spatial dependency.

Mitophagy selectively clears dysfunctional mitochondria. In classical PTEN-induced kinase 1 (PINK1)/Parkin dependent mitophagy, loss of mitochondrial membrane potential leads to PINK1 accumulation on the outer mitochondrial membrane, recruiting and activating Parkin. Activated Parkin ubiquitinates outer mitochondrial membrane proteins to mark damaged mitochondria (Koyano et al., 2014), facilitating their engulfment by autophagosomes and subsequent autolysosomal degradation (Matsuda et al., 2010; Narendra et al., 2008). This process limits sustained ROS generation by dysfunctional mitochondria and maintains cellular energy and redox homeostasis. Notably, PINK1/Parkin is not the sole mitophagy pathway; receptor dependent mechanisms also participate in clearing damaged mitochondria.

Evaluating autophagic status requires distinguishing among autophagy initiation, autophagosome formation, autophagosome-lysosome fusion, and substrate degradation. Increased microtubule-associated protein 1A/1 B-light chain 3 II (LC3-II) levels can reflect either enhanced autophagosome formation or impaired degradation; sequestosome 1 (p62) accumulation must be interpreted alongside transcriptional changes and lysosomal degradative capacity. Static markers such as Akt/mTOR activity or LC3, p62, PINK1, and Parkin levels are insufficient to judge autophagic function; comprehensive analysis requires evaluating lysosomal status, substrate degradation, and dynamic autophagic flux.

#### 2.2.4 Lipid peroxidation and ferroptosis

Ferroptosis is a form of regulated cell death driven by iron-dependent membrane phospholipid peroxidation, characterized by phospholipid hydroperoxide accumulation and an imbalanced glutathione peroxidase 4 (GPX4) defense system (Dixon et al., 2012). Solute carrier family 7 member 11 (SLC7A11) mediated cystine uptake provides substrate for glutathione (GSH) synthesis, and GSH dependent GPX4 reduces membrane phospholipid hydroperoxides to limit lipid peroxidation and ferroptosis (Dixon et al., 2012; Yang et al., 2014). Acyl-CoA synthetase long-chain family member 4 (ACSL4) and lysophosphatidylcholine acyltransferase 3 (LPCAT3) promote polyunsaturated fatty acid incorporation into membrane phospholipids through fatty acid activation and phospholipid remodeling, modulating ferroptosis susceptibility (Doll et al., 2017; Dixon et al., 2015). Although ferroptosis manifests as uncontrolled membrane lipid peroxidation, substrate generation and clearance engage multiple organelles. The ER participates in phospholipid synthesis and remodeling, mitochondria supply energy and reducing equivalents, lysosomes manage iron metabolism, peroxisomes generate polyunsaturated fatty acid containing ether phospholipids, and lipid droplets regulate free fatty acid storage and lipid peroxidation substrate availability (Zou et al., 2020; Olzmann and Carvalho, 2019). Thus, ferroptosis represents a multi organellar imbalance in lipid redox state.

In renal ischemia-reperfusion injury and tubular cell hypoxia/reoxygenation models, salidroside enhances PI3K/Akt signaling, restores GPX4, and suppresses ACSL4 and lipid peroxidation; PI3K/Akt inhibitors partially reverse these protective effects, implicating the pathway in tubular anti-ferroptotic responses (Tang et al., 2023). In diabetic kidney disease models, chicoric acid promotes progestin and adipoQ receptor family member 3 (PAQR3) ubiquitination and attenuates the interaction between PAQR3 and the PI3K catalytic subunit p110α, enhancing PI3K/Akt signaling to alleviate ferroptosis (Zhang et al., 2024). Overexpression of ribonucleotide reductase regulatory subunit M2 (RRM2) activates PI3K/Akt/Nrf2 signaling in high-glucose-stimulated human kidney 2 (HK-2) cells, restoring SLC7A11, GSH, and GPX4 antioxidant defenses while decreasing ROS, Fe^2+^, and lipid peroxidation (Gao et al., 2025). Apigenin was reported to attenuate angiotensin II-induced hypertensive renal injury through a LY294002-sensitive PI3K/Akt-associated mechanism (Zhang et al., 2025). However, inconsistencies in the reported ferroptosis-related markers warrant cautious interpretation of its anti-ferroptotic effects.

**FIGURE 2:**
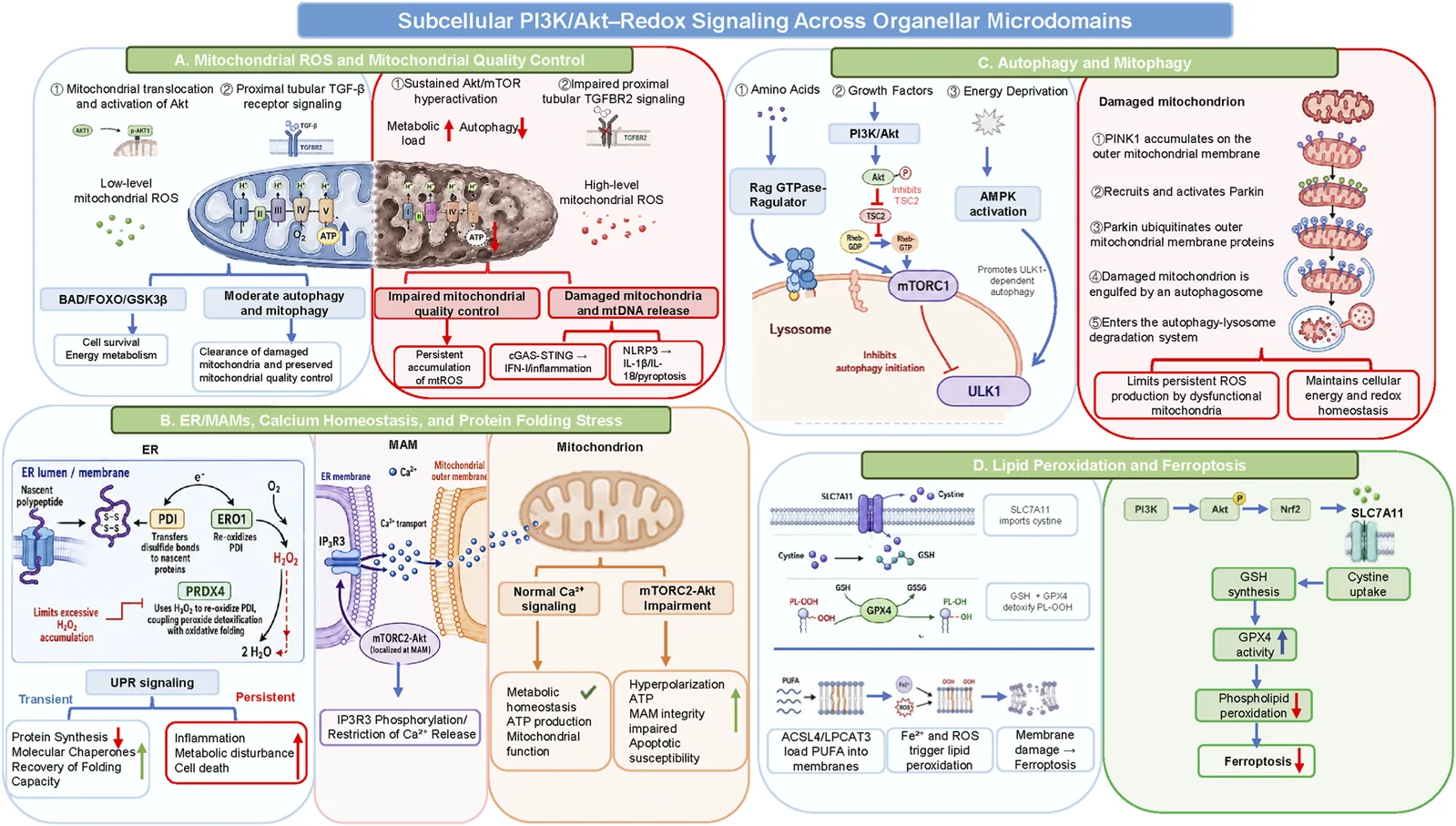
Subcellular PI3K/Akt-redox signaling across organellar microdomains. **(A)** Mitochondrial AKT1 translocation, moderate autophagy, and intact mitochondrial quality control support oxidative phosphorylation and cell survival. Sustained Akt-mTOR activity or impaired mitochondrial quality control promotes mitochondrial reactive oxygen species (mtROS) accumulation and the release of mtDNA or other cellular dsDNA. Cytosolic DNA activates cGAS-STING-dependent type I interferon and inflammatory signaling, whereas NLRP3 activation promotes IL-1β/IL-18 release and pyroptosis. **(B)** In the ER, the ERO1-PDI-PRDX4 system couples oxidative protein folding to H_2_O_2_ metabolism. At mitochondria-associated ER membranes, mTORC2-Akt-dependent phosphorylation of IP3R3 limits excessive Ca^2+^ transfer to mitochondria and helps maintain mitochondrial homeostasis. **(C)** Lysosomal mTORC1 integrates amino acid and growth-factor signals and suppresses ULK1-dependent autophagy, whereas AMPK promotes autophagy during energy deprivation. PINK1/Parkin-mediated mitophagy removes damaged mitochondria and limits persistent ROS production. **(D)** Ferroptosis is governed by the balance between ACSL4/LPCAT3-dependent phospholipid remodeling and SLC7A11-GSH-GPX4-mediated antioxidant defense. PI3K/Akt/Nrf2 signaling can reinforce this defense by supporting cystine uptake, glutathione synthesis, and GPX4 activity.

In summary, ROS source, duration, and subcellular localization determine the input properties of PI3K/Akt signaling, while the homeostasis of mitochondria, ER/MAMs, autolysosomal systems, and lipid-metabolizing organelles shapes its downstream outputs. Moderate, reversible PI3K/Akt signaling supports energy metabolism, proteostasis, and damaged component clearance; sustained imbalance can trigger mitochondrial ROS accumulation, ER stress, autophagic flux blockade, and uncontrolled lipid peroxidation. These mechanisms provide a shared regulatory foundation across renal cell types, the precise manifestations of which depend on cell type, disease stage, and injury severity. Figure 2 illustrates the molecular and organellar mechanisms of PI3K/Akt redox regulation.

## 3 Functional divergence of PI3K/Akt across distinct renal cell states in CKD

### 3.1 Podocytes and the glomerular filtration barrier: the mTOR homeostatic window

Podocyte studies provide direct renal genetic evidence illustrating the dual, bidirectional effects of the PI3K/Akt/mTOR signaling axis. Inoki et al. demonstrated that mTORC1 activity is markedly elevated in podocytes of diabetic animals. Podocyte-specific deletion of tuberous sclerosis 1 (*Tsc1*) led to sustained mTORC1 hyperactivation, inducing proteinuria, glomerular basement membrane (GBM) thickening, mesangial expansion, podocyte hypertrophy and loss, and epithelial-mesenchymal transition (EMT)-like phenotypic changes in non-diabetic mice. Conversely, genetically attenuating mTORC1 activity in diabetic mice by reducing podocyte regulatory-associated protein of mTOR (*Rptor*) gene dosage alleviated foot process effacement, GBM thickening, mesangial expansion, and proteinuria (Inoki et al., 2011). These findings establish that sustained mTORC1 hyperactivation in podocytes is sufficient to drive diabetic kidney disease (DKD)-like filtration barrier injury.

Gödel et al. (2011) further demonstrated that podocyte mTOR activity must be maintained within a tight physiological range. Podocyte-specific deletion of *Rptor* resulted in early-onset proteinuria and progressive glomerulosclerosis. Concurrent deletion of both *Rptor* and *Rictor* exacerbated widespread foot process effacement, proteinuria, and glomerulosclerosis. Although isolated *Rictor* deletion produced no obvious baseline renal phenotype, it increased transient proteinuria under albumin overload, suggesting that mTORC2 participates in podocyte adaptation to external stress. Additionally, loss of podocyte insulin receptor/PI3K/Akt signaling causes cytoskeletal abnormalities, foot process injury, and proteinuria (Welsh et al., 2010); loss of AKT serine/threonine kinase 2 gene (*Akt2)* compromises podocyte survival under CKD-related stress (Canaud et al., 2013). Podocytes also exhibit high basal autophagy activity, and impaired autophagy increases their susceptibility to aging and proteinuric injury (Hartleben et al., 2010).

Podocyte injury also demonstrates clear subcellular and organellar dependence. Hyperactivation of mTORC1 induces ER stress, mislocalization of slit diaphragm proteins, and fibroblast-like phenotypic transitions (Inoki et al., 2011). The chemical chaperone 4-phenylbutyric acid (4-PBA) downregulated 78-kDa glucose-regulated protein (GRP78) expression and partially attenuated podocyte phenotypic transition and cell loss, but failed to restore normal membrane localization of nephrin or significantly improve proteinuria (Inoki et al., 2011). This indicates that ER stress constitutes an important branch of mTORC1-mediated podocyte injury. Proteinuria and high glucose themselves induce renal ER stress (Lindenmeyer et al., 2008), while mTOR signaling imbalance alters the expression and localization of slit diaphragm proteins such as nephrin and podocin and disrupts the podocyte actin cytoskeleton (Inoki et al., 2011; Vollenbröker et al., 2009). In diabetic environments, mTOR/NADPH oxidase 4 (NOX4) signaling increases podocyte ROS production and promotes cell loss (Eid et al., 2013); high glucose can also induce structural and oxidative phosphorylation abnormalities in mitochondria via cyclin-dependent kinase 5 (Cdk5) - sirtuin 1 (Sirt1) mechanisms, resulting in increased ROS, decreased ATP production, and loss of slit diaphragm proteins (Wang et al., 2021). Furthermore, ATP-binding cassette transporter A1 (ABCA1) deficiency and cardiolipin accumulation link aberrant lipid metabolism to podocyte mitochondrial dysfunction, accelerating DKD progression (Ducasa et al., 2019).

These studies provide a mechanistic basis for understanding the dual renal effects of mTOR inhibitors. In animal models with mTORC1 hyperactivation, such as DKD, moderate attenuation of mTORC1 activity alleviates podocyte injury and proteinuria (Inoki et al., 2011; Gödel et al., 2011). However, across various proteinuric nephropathy models, rapamycin exerts either protective or detrimental effects on podocytes depending on administration timing, dosage, disease etiology, and baseline podocyte state (Torras et al., 2009). Clinical studies indicate that some renal transplant recipients treated with rapamycin develop new-onset or worsened proteinuria; higher drug exposure is associated with new-onset focal segmental glomerulosclerosis (FSGS) and reduced expression of podocyte differentiation markers (Letavernier et al., 2007). Another cohort study revealed that sirolimus-associated proteinuria is dose-dependent and accompanied by reduced expression of slit diaphragm-associated proteins, including nephrin, podocin, and CD2-associated protein (CD2AP) (Stallone et al., 2011). Therefore, targeting PI3K/Akt/mTOR in CKD should aim to correct aberrant activity and restore podocyte homeostasis rather than achieve prolonged, systemic, indiscriminate mTOR inhibition.

### 3.2 Tubular stress: acute adaptation versus chronic maladaptive repair

Proximal tubular epithelial cells represent a primary target of redox stress in CKD. Their active transport functions depend heavily on fatty acid oxidation and mitochondrial oxidative phosphorylation, rendering them highly sensitive to ischemia, protein overload, uremic toxin accumulation, and metabolic disturbances (Liu et al., 2018; Kang et al., 2015). These stimuli induce mitochondrial dysfunction, insufficient ATP production, ROS accumulation, and impaired cellular quality control, subsequently altering tubular cell survival, differentiation status, and paracrine phenotypes. Tubular PI3K/Akt signaling varies with the phase of injury, Akt isoform, subcellular localization, and downstream effector branch.

During acute injury, moderate PI3K/Akt activation functions primarily in antioxidant defense, anti-apoptosis, and mitochondrial homeostasis. Martin et al. established the foundational mechanism in non-renal cells whereby PI3K/Akt regulates Nrf2-dependent antioxidant response element (ARE) activation and heme oxygenase-1 (HO-1) expression (Martin et al., 2004). In tubule-specific investigations, Lin et al. (Lin et al., 2021) found that ischemia-reperfusion induces AKT1 translocation to tubular mitochondria, where it becomes activated; inhibiting mitochondrial AKT1 exacerbates oxidative stress, tubular injury, and subsequent fibrosis, whereas enhancing mitochondrial AKT1 activity preserves oxidative phosphorylation and cell survival. Furthermore, Li et al. (Li et al., 2024) showed that family with sequence similarity 3 member A (FAM3A) reduces mitochondrial ROS accumulation through the P2Y purinergic receptor 1 (P2Y1)/PI3K/Akt/Nrf2 axis and suppresses NLR family pyrin domain-containing 3 (NLRP3)–caspase-1–gasdermin D (GSDMD)-mediated tubular pyroptosis in ischemic AKI. Beyond PI3K/Akt, TEA domain transcription factor 1 (TEAD1) provides an additional transcriptional mechanism supporting tubular stress adaptation. In cisplatin-induced acute kidney injury, proximal tubule-specific *Tead1* deletion aggravated mitochondrial bioenergetic impairment, reactive oxygen species accumulation, necroptosis, inflammation, tubular injury, and kidney dysfunction. Mechanistically, TEAD1 interacted with peroxisome proliferator-activated receptor gamma coactivator 1-alpha (PGC-1α) to maintain mitochondrial function (Tran et al., 2025). This protective tubular role contrasts with the profibrotic TEAD-associated transcriptional program in interstitial fibroblasts discussed below, further emphasizing the compartment-dependent functions of TEAD-family signaling.

As injury persists, Akt signaling can shift from stress adaptation to maladaptive repair. Kattla et al. (Kattla et al., 2008) observed in tubular epithelial cells and DKD models that transforming growth factor beta 1 (TGF-β1) activates PI3K/Akt/GSK3β signaling, inducing a loss of epithelial markers and an increase in mesenchymal markers. AKT serine/threonine kinase 2 (AKT2) was also shown to participate in TGF-β1-induced tubular EMT-like dedifferentiation via GSK3β inactivation and Snail stabilization (Lan et al., 2014). In AKI-to-CKD transition models, AKT1 activation is more sustained than AKT2 or AKT3 activation. *Akt1* deficiency suppresses GSK3β/Snail and β-catenin signaling, reduces tubular dedifferentiation and apoptosis, and attenuates interstitial fibrosis (Kim et al., 2021; Kim et al., 2023). The protective role of mitochondrial AKT1 during the acute phase, contrasted with the pro-fibrotic effect of sustained AKT1 activation during the chronic phase, underscores that AKT1 function is tightly constrained by subcellular localization and signal duration.

Recent evidence identifies SRY-box transcription factor 4 (SOX4) as an additional transcriptional regulator of maladaptive tubular remodeling. Using global, tubular epithelial cell-specific, and fibroblast-specific loss-of-function approaches in obstructive, ischemia-reperfusion, and folic acid-induced kidney fibrosis models, Du et al. (Du et al., 2026) demonstrated that SOX4 promotes tubular epithelial dedifferentiation, fibroblast activation, and renal fibrosis. Mechanistically, SOX4 facilitated TGF-β1-SMAD family member 3 (Smad3) signaling, whereas SOX4 depletion preserved epithelial characteristics and attenuated profibrotic remodeling. These findings place SOX4-Smad3 alongside the sustained AKT1/AKT2-GSK3β-Snail pathway within the broader transcriptional network governing maladaptive tubular repair, although direct regulation of SOX4 by PI3K/Akt in injured renal tubules has not yet been established.

Autophagy and mitochondrial quality control serve as critical bridges linking Akt/mTOR signaling, redox homeostasis, and tubular injury outcomes. Tubule-specific autophagy-related 5 gene (*Atg5)* deletion causes the accumulation of abnormal proteins and damaged mitochondria and aggravates tubular apoptosis and kidney dysfunction after ischemia-reperfusion, demonstrating the protective role of basal autophagy during acute stress (Kimura et al., 2011). Under persistent protein overload or metabolic stress, the autophagic system may transition from effective quality control to flux impairment. Proteinuria impairs normal autophagosome processing and lysosomal degradation in proximal tubules, preventing the clearance of abnormal proteins and damaged organelles, thereby linking protein overload to tubular atrophy and interstitial fibrosis (Nolin et al., 2016). In DKD, intermediate-conductance calcium-activated potassium channel KCa3.1-mediated -mediated aberrant PI3K/Akt/mTOR signaling causes tubular autophagic dysfunction (Huang et al., 2016). Because mTORC1 primarily suppresses autophagy initiation, elevated Akt/mTOR signaling typically reduces autophagy induction; however, in complex disease environments, autophagic status is also influenced by lysosomal acidification, membrane fusion, substrate load, and energy supply.

Autophagy may also drive maladaptive remodeling during chronic injury. In unilateral ureteral obstruction (UUO) models, persistent tubular autophagy activation is accompanied by tubular cell death, inflammatory mediator release, and interstitial fibrosis; pharmacological inhibition of autophagy or tubule-specific disruption of autophagy-related 7 gene *(Atg7)* attenuates these pathological changes, suggesting that sustained autophagy can promote fibrogenesis in specific obstructive injury contexts (Livingston et al., 2016). Yuan et al. (Yuan et al., 2021) observed upregulation of tubular transcription factor EB (TFEB) and autophagy-related genes in adenine-induced CKD mice; in normal rat kidney epithelial cell line 52 E (NRK-52 E) cells, trehalose-induced TFEB activation increased autophagy induction, cell death, and interleukin-6 (IL-6) release. These findings suggest that enhanced autophagy-related transcriptional programs may represent a compensatory response to organellar damage and metabolic stress, but can also participate in maladaptive remodeling in specific chronic settings.

In summary, the biological significance of tubular PI3K/Akt signaling depends on the injury phase, Akt isoforms, subcellular localization, and downstream branches. During the acute phase, mitochondrial AKT1 translocation and activation, together with Nrf2-related responses, support cell survival and redox homeostasis, whereas persistent AKT1/AKT2-GSK3β-Snail, SOX4-Smad3, Akt/mTOR, and autophagy-related imbalances promote tubular dedifferentiation, cell death, and profibrotic repair.

### 3.3 Microvascular endothelial homeostasis and the hypoxic-inflammatory niche

CKD progression is frequently accompanied by renal microvascular endothelial injury, peritubular capillary rarefaction, and tissue hypoxia (Kida et al., 2014). Ischemic AKI-to-CKD models demonstrate that microvascular endothelial cell injury and capillary loss persist into the late phase of injury, closely correlating with renal tissue hypoxia and interstitial fibrosis; inhibiting endothelial cell apoptosis attenuates microvascular rarefaction and long-term renal fibrosis (Yang et al., 2018). In uremic mice, CKD causes microvascular network disruption, reduced tissue oxygen extraction, and impaired hypoxia-driven angiogenesis (Prommer et al., 2018). Microvascular endothelium serves both as a target of CKD injury and as a key component regulating perfusion, hypoxia, and the fibrotic microenvironment.

Under physiological conditions, PI3K/Akt signaling is an essential regulatory pathway for maintaining endothelial homeostasis. Dimmeler et al. (Dimmeler et al., 1999) and Fulton et al. (Fulton et al., 1999) demonstrated that Akt directly phosphorylates endothelial nitric oxide synthase (eNOS) at Ser1177, enhancing eNOS activity and nitric oxide (NO) production. Akt-eNOS-NO signaling helps preserve vasodilation, microvascular perfusion, and endothelial anti-adhesive and anti-thrombotic functions (Förstermann and Sessa, 2012). Thus, retaining moderate Akt-eNOS signaling in renal microvessels during CKD may help maintain local blood flow and oxygen delivery while restricting endothelial activation and inflammatory cell recruitment. However, CKD-associated hyperglycemia, uremic toxin accumulation, and chronic oxidative stress can impair this protective output. High glucose increases *O*-GlcNAcylation of eNOS near its Akt phosphorylation site, diminishing eNOS phosphorylation and enzymatic activity, thereby reducing NO production (Du et al., 2001). The uremic toxin indoxyl sulfate induces ROS generation and NF-κB activation in vascular endothelial cells, promoting the expression of intercellular adhesion molecule-1 (ICAM-1) and monocyte chemoattractant protein-1 (MCP-1) (Tumur et al., 2010). CKD-relevant urea concentrations increase mitochondrial ROS and inhibit glyceraldehyde-3-phosphate dehydrogenase (GAPDH) in endothelial cells, thereby activating protein kinase C, hexosamine, and advanced glycation end-product-related pathways. Urea-induced ROS also enhance NF-κB-dependent MCP-1 and vascular cell adhesion molecule 1 (VCAM-1) expression and reduce prostacyclin synthase activity, promoting a proinflammatory and proatherogenic endothelial phenotype (D'Apolito et al., 2015). In endothelial cell models, NADPH oxidase-derived ROS do not merely cause nonspecific damage; instead, they selectively regulate vascular endothelial growth factor (VEGF)-induced PI3K/Akt/FOXO and p38 mitogen-activated protein kinase (p38 MAPK) signaling, with minimal effects on the extracellular signal-regulated kinase 1/2 (ERK1/2) branch (Abid et al., 2007).

Therefore, endothelial signaling in CKD is better understood as a dynamic imbalance between the Akt-eNOS-NO homeostatic module and the ROS-NF-κB inflammatory module. Physiological Akt-eNOS activity supports vasodilation, endothelial survival, and microvascular stability. In contrast, persistent NOX- or mitochondria-derived ROS generated in the uremic environment reduce NO bioavailability and favor NF-κB-dependent adhesion, barrier dysfunction, and vascular remodeling. Given that distinct NOX isoforms, subcellular localizations, and generated ROS may exert differential signaling effects, elevated NOX4 expression cannot be directly equated with pathological oxidative stress.

### 3.4 Myeloid cells and immunometabolism

CKD-associated inflammation is driven by complex interactions among renal parenchymal cells, endothelial cells, myeloid cells, and interstitial cells (Lake et al., 2023). Among these, macrophages serve as pivotal cellular nodes bridging tissue injury, inflammatory responses, and repair/remodeling, comprising tissue-resident macrophages and monocyte-derived macrophages, which differ in lineage origin and functional states (Liu F. et al., 2020). Single-cell and spatial omics studies have further revealed that myeloid cells in CKD kidneys exhibit continuous and heterogeneous state transitions, encompassing programs such as inflammatory responses, phagocytic clearance, antigen presentation, lipid metabolism, and tissue remodeling (Conway et al., 2020). Consequently, the traditional classically activated (M1) versus alternatively activated (M2) macrophage dichotomy fails to accurately capture myeloid cell states in CKD, making classification based on cell lineage, spatial localization, and functional gene modules far more appropriate.

The role of PI3K/Akt in myeloid cells is markedly context-dependent. This pathway supports macrophage survival and phagocytic clearance of apoptotic cells (efferocytosis), thereby facilitating inflammatory resolution and tissue repair (Linton et al., 2019). Phosphoinositide 3-kinase gamma (PI3Kγ) is primarily involved in chemotactic migration and myeloid cell recruitment, and its pathological consequences depend on recruitment timing and cellular states (Hirsch et al., 2000). Myeloid-specific PTEN deletion removes PTEN-mediated negative regulation of PI3K/Akt signaling, thereby exacerbating angiotensin II-induced macrophage infiltration, renal inflammation, and interstitial fibrosis (An et al., 2022). Alongside PI3K/Akt signaling, myeloid cell states are shaped by cytokine-responsive transcriptional and epigenetic networks. Genetic deficiency of signal transducer and activator of transcription 6 (STAT6) reduces alternative macrophage polarization, myeloid fibroblast accumulation, and renal fibrosis in folic acid nephropathy (Jiao et al., 2021a), whereas pharmacological STAT6 inhibition produces similar effects in obstructive and folic acid-induced nephropathy (Jiao et al., 2021b). Jumonji domain-containing protein 3 (JMJD3) promotes interferon regulatory factor 4 (IRF4) expression and profibrotic macrophage activation, whereas genetic or pharmacological inhibition of JMJD3 attenuates these responses in obstructive nephropathy (An et al., 2023). Independent evidence further links the interleukin-4 (IL-4)-JMJD3-IRF4 axis to macrophage-to-myofibroblast transition and ECM deposition (Liang et al., 2022). Together, these findings suggest that STAT6 and JMJD3-IRF4 operate alongside PI3K/Akt to promote specific profibrotic myeloid states, rather than forming a single established linear signaling cascade.

Inflammatory receptors such as toll-like receptor 4 (TLR4) can simultaneously engage PI3K/Akt, NF-κB, and mitogen-activated protein kinase (MAPK) signaling. The effect of PI3K/Akt on inflammatory output varies with the stimulus and cellular state and may either enhance or limit cytokine production (Ojaniemi et al., 2003; Guha and Mackman, 2002). NADPH oxidase 2 (NOX2)-derived ROS selectively regulate the expression of inflammatory genes including Interleukin 1 beta gene (*IL1B)*, Interleukin 6 gene (*IL6)*, and chemokines in macrophages (Bae et al., 2009); mitochondrial damage, increased mitochondrial ROS, and oxidized mtDNA release promote NLRP3 inflammasome activation and the secretion of interleukin 1 beta (IL-1β) and interleukin 18 (IL-18) (Zhou et al., 2011; Nakahira et al., 2011). Inflammasome activation comprises an NF-κB-dependent priming phase involving the induction of NLRP3 and pro-IL-1β, followed by an activation phase triggered by signals such as potassium efflux, lysosomal injury, mitochondrial dysfunction, and oxidized DNA. Canonical NLRP3 inflammasome assembly requires NIMA-related kinase 7 (NEK7) and activates caspase-1, which processes IL-1β and IL-18 and cleaves gasdermin D (GSDMD) to induce pyroptosis. Non-canonical caspase-4/5/11 signaling and DNA-responsive inflammasomes such as absent in melanoma 2 (AIM2) provide additional routes through which intracellular danger signals sustain inflammatory cell death (Li et al., 2025). Nrf2 constitutes a vital negative regulatory node in the intracellular redox-inflammatory coupling network of myeloid cells. Kobayashi et al. found that Nrf2 directly suppresses the transcription of pro-inflammatory genes such as *IL6* and *IL1B* in macrophages, an effect that is not entirely dependent on ROS scavenging (Kobayashi et al., 2016). In the Akita model of diabetic kidney disease, global Nrf2 deficiency exacerbates oxidative damage, macrophage infiltration, inflammation, and fibrosis, whereas hypomorphic kelch-like ECH-associated protein 1 (Keap1)-mediated mild Nrf2 activation alleviates tubular injury and inflammatory gene expression (Liu et al., 2022). Because this study was not conducted in a myeloid cell-specific model, the observed protective effects cannot be attributed solely to endogenous myeloid Nrf2.

Ultimately, these intracellular signaling events influence disease progression through bidirectional crosstalk between myeloid cells and renal tubular or interstitial cells. Following ischemia-reperfusion, IL-1β and H_2_O_2_ induce renal tubular epithelial cells to sequentially express chemokines such as macrophage inflammatory protein 1 alpha (MIP-1α) and C-C motif chemokine ligand 2 (CCL2)/MCP-1, promoting macrophage recruitment (Furuichi et al., 2006); tubular-derived colony-stimulating factor 1 (CSF-1) facilitates tissue repair through macrophage-dependent actions and tubular autocrine/paracrine mechanisms, whereas colony-stimulating factor 1 receptor (CSF-1R) blockade exacerbates tubular damage and fibrosis (Menke et al., 2009). During the chronic fibrotic stage, macrophages promote fibrogenesis by secreting vitronectin, which activates integrin αvβ5/Src signaling in fibroblasts (Peng et al., 2023); inflammatory fibroblasts can also recruit monocytes and induce the formation of folate receptor beta (FOLR2^+^) macrophages, which in turn drive fibroblast transition into ECM-secreting myofibroblasts via wingless-related integration site signaling pathway (WNT)/β-catenin signaling (Cohen et al., 2024). Thus, the interactions between myeloid cells and renal parenchymal/interstitial cells display clear stage dependency, shifting from injury clearance and tissue repair during the acute phase to inflammation maintenance and fibrotic remodeling in chronic stages.

### 3.5 The fibrotic microenvironment: Fibroblasts, pericytes, and ECM remodeling

Renal interstitial fibrosis is driven by cell state transitions, paracrine signaling, and ECM remodeling. Genetic lineage tracing studies show that Forkhead box D1 (*Foxd1*)-derived pericytes and perivascular interstitial cells proliferate and acquire a myofibroblast phenotype after renal injury, serving as major sources of scar-forming cells (Humphreys et al., 2010). Human kidney single-cell transcriptomic studies further reveal functionally heterogeneous fibroblast and pericyte subpopulations in fibrotic kidneys, whose activation states closely correlate with injured tubular, vascular, and immune microenvironments (Lake et al., 2023; Abedini et al., 2024). Renal fibrosis is therefore best understood as a pathological niche comprising injured epithelial cells, myeloid cells, pericytes, fibroblasts, and myofibroblasts rather than as an autonomous process driven by a single cell population.

In this microenvironment, both mTORC1 and mTORC2 participate in renal fibroblast activation. Studies demonstrate that Rheb/mTORC1 signaling is augmented in interstitial myofibroblasts of fibrotic kidneys, and inhibiting this pathway attenuates TGF-β1-induced fibroblast activation and renal interstitial fibrosis (Jiang et al., 2013). Li et al. (Li et al., 2015) further proved that Rictor/mTORC2 signaling mediates TGF-β1-induced renal fibroblast activation; blocking Rictor/mTORC2 reduces fibrotic phenotypes including α-smooth muscle actin (α-SMA), fibronectin, and collagen expression, alleviating experimental renal fibrosis. Gui et al. (Gui et al., 2018) found that Yes-associated protein/transcriptional coactivator with PDZ-binding motif (YAP/TAZ) serves as a vital downstream mediator of mTORC2 profibrotic effects; inhibiting Rictor/mTORC2 or YAP/TAZ weakens TGF-β1-induced fibroblast activation. Because YAP/TAZ lack intrinsic sequence-specific DNA-binding activity, their nuclear transcriptional effects depend largely on TEA domain (TEAD) family transcription factors. In unilateral ureteral obstruction kidneys, expression of TEA domain family members 1-4 (TEAD1-4) increased together with Akt phosphorylation at serine 473 and YAP/TAZ activation in interstitial myofibroblasts. Fibroblast/pericyte-specific *Rictor* deletion or pharmacological mTOR inhibition reduced TEAD1-4, connective tissue growth factor (CTGF), and ankyrin repeat domain 1 (ANKRD1) expression, accompanied by decreased fibroblast activation and ECM deposition (Gui et al., 2018). These findings support an mTORC2-YAP/TAZ-TEAD transcriptional module linking TGF-β1 signaling to profibrotic gene expression. However, because TEAD1-4 were evaluated collectively, the specific and non-redundant requirement for TEAD1 in mTORC2-driven renal fibroblast activation remains to be established.

Beyond TGF-β1, CKD-associated metabolites also act on renal medullary fibroblasts via Akt/mTOR signaling. TMAO increases α-SMA expression, cell proliferation, and total collagen production. Pharmacological inhibition experiments suggest that protein kinase R-like endoplasmic reticulum kinase (PERK)/Akt/mTOR signaling contributes to TMAO-induced fibroblast proliferation and collagen production, whereas NLRP3 and caspase-1 appear to contribute primarily to the proliferative response (Kapetanaki et al., 2021). These findings indicate that mTORC1 primarily governs cell growth, protein synthesis, and anabolism, whereas mTORC2 regulates the cytoskeleton, survival, and profibrotic transcriptional programs through Akt and YAP/TAZ branches; together, they link growth factors and uremic metabolic stimuli to ECM remodeling.

Pericyte-to-myofibroblast transition represents another major mechanism of renal fibrosis. Previous studies showed that platelet-derived growth factor receptor (PDGFR) signaling promotes pericyte detachment from microvessels, driving obstructive and post-ischemic renal fibrosis (Chen et al., 2011). In primary renal pericytes, TGF-β1 increases PI3K, Akt, and mTOR phosphorylation together with hexokinase II(HKII) expression, glucose consumption, and glycolytic activity. Inhibiting PI3K/Akt/mTOR or glycolysis attenuates pericyte-to-myofibroblast transition, whereas HKII overexpression partially restores this process (Chen et al., 2023). This study provides direct cellular mechanistic evidence for PI3K/Akt/mTOR-mediated pericyte state transitions via metabolic reprogramming, though its findings rely primarily on *in vitro* primary pericytes and lack causal validation in cell-specific animal models.

**FIGURE 3:**
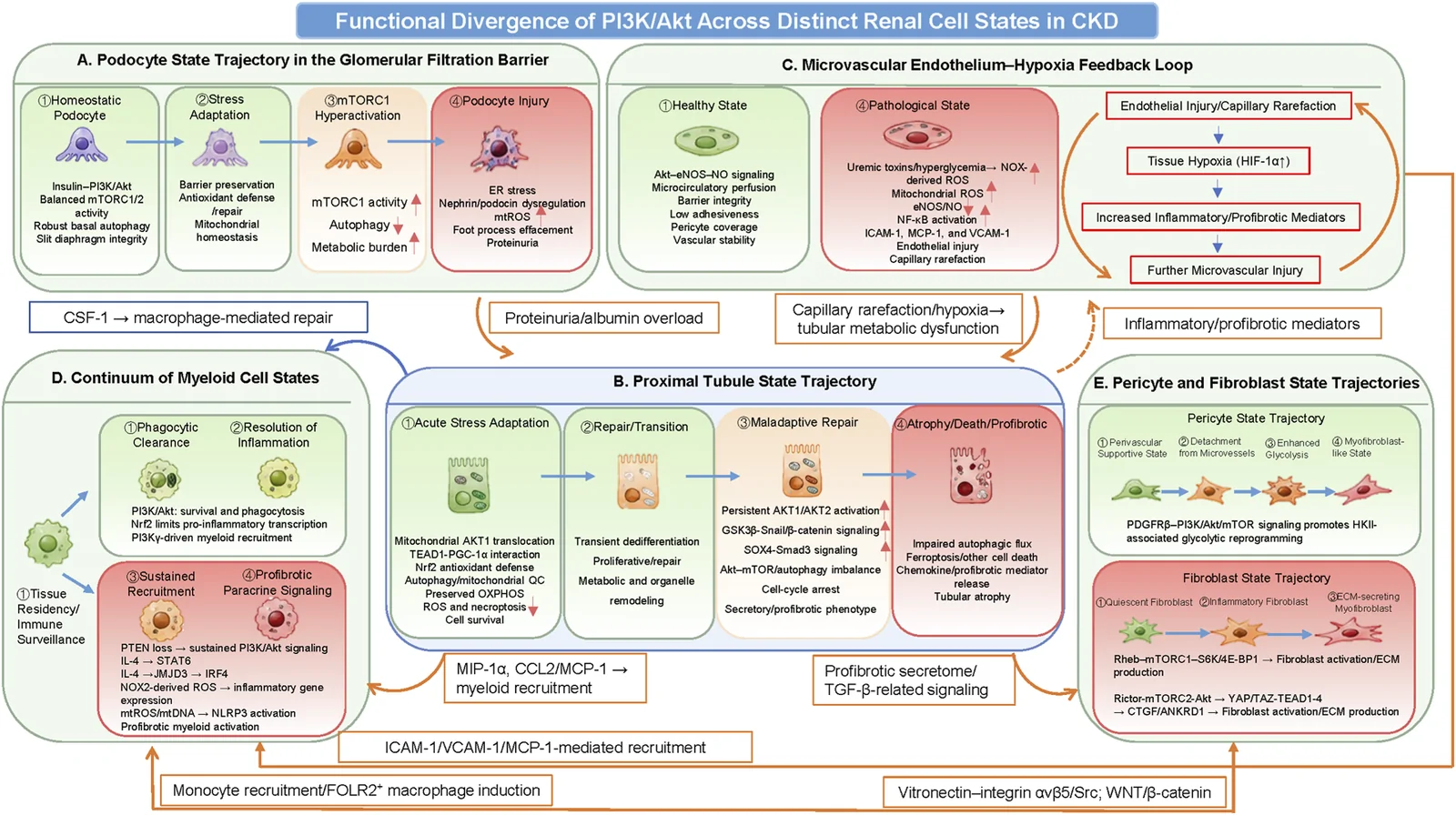
Cell-state-dependent functions of PI3K/Akt signaling in CKD. **(A)** In podocytes, physiological insulin-PI3K/Akt-mTOR signaling and basal autophagy maintain slit-diaphragm integrity and support adaptation to stress. Sustained mTORC1 activation promotes autophagy inhibition, ER stress, mitochondrial ROS accumulation, foot-process effacement, and proteinuria. **(B)** Proximal tubular cells progress from acute stress adaptation and transient repair to maladaptive repair, atrophy, and profibrotic signaling. During acute injury, mitochondrial AKT1 and Nrf2 support survival and redox homeostasis, while TEAD1-PGC-1α helps preserve mitochondrial function and limits ROS accumulation and necroptosis. During persistent injury, AKT1/AKT2-GSK3β-Snail, TGF-β1-SOX4-Smad3, and Akt-mTOR-associated autophagy imbalance promote dedifferentiation and secretory reprogramming. **(C)** Endothelial Akt-eNOS-NO signaling maintains microvascular perfusion and barrier function. Persistent oxidative and inflammatory stress causes endothelial injury, capillary rarefaction, tissue hypoxia, and a self-reinforcing profibrotic feedback loop. **(D)** Myeloid cells occupy a continuum from tissue surveillance, phagocytic clearance, and inflammatory resolution to sustained recruitment and profibrotic paracrine signaling. IL-4-STAT6 and JMJD3-IRF4 programs act alongside context-dependent PI3K/Akt signaling to promote profibrotic myeloid states. **(E)** Platelet-derived growth factor receptor beta (PDGFRβ)-PI3K/Akt/mTOR-HKII signaling promotes glycolytic reprogramming during pericyte-to-myofibroblast transition. In fibroblasts, Rheb-mTORC1-S6K/4 E-BP1 and Rictor-mTORC2-Akt signaling promote activation and ECM production through distinct downstream branches. The mTORC2 branch engages YAP/TAZ-TEAD1-4 and induces CTGF and ANKRD1. Intercellular signaling among tubular, endothelial, myeloid, and stromal cells reinforces CKD progression.

Integrating current evidence, PI3K/Akt/mTOR regulation within the fibrotic microenvironment can be divided into three tiers: upstream stimuli; signal and metabolic integration; and cellular and ECM outputs. Stimuli such as TGF-β1, platelet-derived growth factor (PDGF), and uremic metabolites activate downstream branches including Rheb/mTORC1-ribosomal protein S6 kinase (S6K)/eukaryotic translation initiation factor 4 E-binding protein 1 (4 E-BP1), Rictor/mTORC2-Akt-YAP/TAZ, and PI3K/Akt/mTOR-HKII. This activation drives fibroblast activation, pericyte transition toward myofibroblast-like states, and the expression of fibrotic genes including actin alpha 2, smooth muscle (*ACTA2)*, collagen type I alpha 1 chain (*COL1A1)*, and fibronectin 1 (*FN1)*, alongside collagen production. Consequently, PI3K/Akt/mTOR serves as a crucial signal integration node linking profibrotic stimuli, cell state transitions, and metabolic reprogramming. Nevertheless, targeted interventions must distinguish between mTORC1 and mTORC2, as well as fibroblasts and pericytes, while accounting for signal intensity and disease stage to avoid equating systemic pathway blockade with cell-specific anti-fibrotic efficacy. Figure 3 illustrates the functional divergence of PI3K/Akt signaling across distinct renal cell states in CKD.

## 4 From spatial mechanisms to clinical translation

### 4.1 Single-cell and spatial omics: From cell state description to pathway activity validation

Traditional CKD research frequently relies on whole-kidney tissue Western blotting or immunohistochemistry to detect p-Akt, phosphorylated mechanistic target of rapamycin (p-mTOR), Nrf2, HO-1, NF-κB, and ECM-related proteins. Although these approaches reveal average pathway changes in kidney tissue, they cannot reliably determine the cellular source or spatial niche of the signal or distinguish protective from pathogenic outputs across cell states. For instance, elevated whole-kidney p-Akt levels may reflect survival and metabolic adaptation in acutely injured tubules, hyperactivated mTORC1 in podocytes, interstitial cell activation, or myeloid cell accumulation. Thus, tissue-averaged pathway alterations cannot be directly equated with specific pathological mechanisms.

Single-cell and spatial omics provide a new evidentiary foundation to resolve this heterogeneity. Lake et al. (Lake et al., 2023) constructed a multimodal human kidney atlas using single-cell, single-nucleus transcriptomics, epigenomics, and spatial imaging, defining 51 main cell types and 28 injury-associated cell states while mapping adaptive repair, maladaptive repair, and degenerative states to specific tissue neighborhoods. Abedini et al. (Abedini et al., 2024) integrated single-cell transcriptomics, chromatin accessibility, and spatial transcriptomics to identify glomerular, tubular, immune, and fibrotic microenvironments, demonstrating that fibrotic microenvironment features correlate with disease progression. These datasets provide a cellular and spatial context for interpreting PI3K/Akt-associated transcriptional changes. However, single-cell RNA sequencing (scRNA-seq) and spatial transcriptomics primarily measure transcript abundance and cannot directly reflect Akt phosphorylation, kinase activity, or the cysteine oxidation state of proteins such as PTEN. Gene scores for PI3K/Akt, Nrf2, NF-κB, mTOR, or ferroptosis serve well to identify candidate states and generate hypotheses, but cannot serve as standalone proof of pathway activation; spatial transcriptomics also has limited capacity to detect post-translational protein modifications.

Validating the spatial topology of PI3K/Akt redox signaling in CKD requires integrating multi-layered evidence. scRNA-seq or single-nucleus RNA sequencing (snRNA-seq) can identify state-specific transcriptional programs in injured tubular, podocyte, endothelial, myeloid, and interstitial cells. Spatial transcriptomics and spatial imaging can map the distribution of these states across atrophic tubular regions, microvascular rarefaction zones, immune infiltration niches, and fibrotic microenvironments. Phosphoproteomics, phospho-specific immunoimaging, or mass spectrometry imaging can measure activation readouts such as p-Akt, phosphorylated GSK3β (p-GSK3β), phosphorylated ribosomal protein S6 (p-S6), phosphorylated FOXO (p-FOXO), phosphorylated eNOS (p-eNOS), and phosphorylated NF-κB p65 (p-p65). Redox proteomics can evaluate cysteine oxidation in PTEN, Keap1, protein tyrosine phosphatases, and metabolic enzymes. Metabolomics and lipidomics provide complementary functional readouts including NADPH, GSH/glutathione disulfide (GSSG), lipid hydroperoxides, 4-hydroxynonenal (4-HNE), polyunsaturated fatty acid-containing phospholipids (PUFA-PLs), and acyl-CoAs. Only by mapping cell identity, spatial location, protein activation, redox modification, and metabolic output to one another can “PI3K/Akt involvement in CKD oxidative stress” be transformed into a testable mechanistic model. Recent advances in low-input phosphoproteomics have begun to resolve phosphorylation events from rare-cell and specialized single-cell samples, although unbiased coverage remains substantially shallower than that achieved in bulk tissues (Wu Q. et al., 2025). Single-cell redox proteomics is less mature because cysteine oxidation is labile and analyte amounts are limited; integrating spatial indexing with compartment-resolved cysteine-oxidation workflows may eventually enable direct mapping of PTEN/Keap1 redox states and Akt/mTOR activity in defined renal cell populations (Kisty et al., 2023).

### 4.2 Translation boundaries of Nrf2 activation and mTOR inhibition

Nrf2 is a crucial effector node connecting PI3K/Akt signaling to antioxidant transcriptional responses. Research indicates that Nrf2 forms heterodimers with small musculoaponeurotic fibrosarcoma protein (sMAF) proteins to bind antioxidant response elements (AREs), inducing the expression of NAD(P)H quinone dehydrogenase 1 (NQO1), glutamate-cysteine ligase catalytic subunit (GCLC), glutamate-cysteine ligase modifier subunit (GCLM), and other antioxidant and detoxifying genes; Keap1 restricts Nrf2 stability and nuclear activity by binding its Nrf2-ECH homology 2 (Neh2) domain (Itoh et al., 1997; Itoh et al., 1999). In diabetic kidney disease and other experimental CKD models, genetic or pharmacological enhancement of Nrf2 signaling generally alleviates oxidative damage and inflammation (Jiang et al., 2010; Zheng et al., 2011). These findings support the biological rationale of Nrf2 as a renal redox regulatory target.

However, clinical translation of Nrf2-targeted therapy has not demonstrated a simple dose-benefit relationship. The 52-Week Bardoxolone Methyl Treatment: Renal Function in CKD/Type 2 Diabetes (BEAM) trial demonstrated sustained increases in estimated glomerular filtration rate (eGFR) in patients with type 2 diabetes and CKD (Pergola et al., 2011). The subsequent Bardoxolone Methyl Evaluation in Patients with CKD and Type 2 Diabetes Mellitus: the occurrence of renal events (BEACON) trial did not reduce the risk of ESKD or cardiovascular death and was terminated early because of excess heart-failure events (de Zeeuw et al., 2013). The Phase 2 Study of Bardoxolone Methyl in Patients with CKD and Type 2 Diabetes (TSUBAKI) enrolled patients without prespecified heart-failure risk factors and demonstrated an increase in measured glomerular filtration rate (GFR) assessed by inulin clearance (Nangaku et al., 2020). The subsequent phase 3 AYAME study (A Phase 3 Study of Bardoxolone Methyl in Patients with Diabetic Kidney Disease) evaluated a composite endpoint of eGFR decline or ESKD (Nangaku et al., 2023). According to sponsor-reported results, AYAME met its primary and key secondary composite eGFR endpoints but did not reduce the incidence of ESKD alone; consequently, development of bardoxolone methyl for diabetic kidney disease in Japan was discontinued (Kyowa Kirin Co, 2023). Collectively, these findings indicate that an on-treatment increase or preservation of eGFR should not be interpreted as evidence of structural kidney repair and must be evaluated alongside hard kidney and cardiovascular outcomes.

Importantly, these clinical outcomes should not be interpreted as evidence that potent Nrf2 activation is inherently harmful. Bardoxolone methyl is a systemic pharmacological intervention whose efficacy and safety are influenced by drug selectivity, tissue distribution, dosing, hemodynamic effects, fluid balance, baseline cardiovascular risk, and endpoint selection. Its clinical results therefore illustrate the translational limitations of systemic Nrf2-directed pharmacology and should not be directly equated with the biological effects of cell-specific Nrf2 genetic modulation.

### 4.3 Precision interventions targeted to cell states, disease stages, and spatial microdomains

Precision targeting of PI3K/Akt-redox signaling in CKD should not aim merely at elevating or reducing overall pathway activity, but rather at modulating pathological cell states within defined disease stages and spatial microdomains. The therapeutic objective should shift from modulating a single pathway to controlling cell state trajectories: preserving essential survival and metabolic adaptation signals during acute injury while preventing their transition into persistent dedifferentiation, inflammatory, and fibrotic programs in chronic stages.

During acute tubular injury, mitochondrial AKT1 translocation and activation, Nrf2 antioxidant responses, and intact autolysosomal quality control maintain oxidative phosphorylation and cell survival. Upon transitioning into the AKI-to-CKD phase, persistent AKT1/AKT2-GSK3β/Snail, β-catenin, and Akt/mTOR signaling can lock cells into maladaptive repair states (Li et al., 2024; Lin et al., 2021; Kim et al., 2021; Kattla et al., 2008; Lan et al., 2014; Kim et al., 2023; Kimura et al., 2011; Nolin et al., 2016; Livingston et al., 2016; Yuan et al., 2021). Thus, interventions must respect temporal windows, avoiding premature suppression of protective signals during early stages while selectively terminating pro-dedifferentiation and pro-fibrotic branches in chronic phases.

The key to podocyte-directed therapy lies in preserving the physiological window between PI3K/Akt/mTOR and autophagy. Basal insulin signaling, mTORC1/mTORC2, and autophagic flux are indispensable for filtration barrier homeostasis, whereas sustained mTORC1 activation induces hypertrophy, ER stress, and proteinuria (Inoki et al., 2011; Gödel et al., 2011; Welsh et al., 2010; Canaud et al., 2013; Hartleben et al., 2010; Lindenmeyer et al., 2008; Vollenbröker et al., 2009; Wang et al., 2021; Ducasa et al., 2019; Torras et al., 2009; Letavernier et al., 2007; Stallone et al., 2011). mTOR inhibitors should therefore not be applied uniformly across proteinuric kidney diseases. Treatment selection should consider disease etiology, baseline pathway activity where measurable, proteinuria severity, and anticipated drug exposure.

Microvascular and immune interventions should emphasize decoupling protective from pathogenic branches. Endothelial-directed strategies should preserve Akt-eNOS-NO-dependent perfusion and barrier function (Dimmeler et al., 1999; Fulton et al., 1999; Förstermann and Sessa, 2012) while limiting excessive NOX- or mitochondria-derived ROS and NF-κB-dependent inflammation (Du et al., 2001; Tumur et al., 2010; D'Apolito et al., 2015; Abid et al., 2007). Myeloid-targeted strategies should preserve efferocytosis and inflammatory resolution (Linton et al., 2019; Guha and Mackman, 2002; Kobayashi et al., 2016; Menke et al., 2009), while limiting sustained cell recruitment and pro-fibrotic paracrine signaling (Hirsch et al., 2000; An et al., 2022; Furuichi et al., 2006; Peng et al., 2023; Cohen et al., 2024). Selective interventions should target specific receptors, PI3K isoforms, ROS sources, or intercellular communication axes rather than broadly suppressing entire signaling networks.

In the fibrotic stage, branches including Rheb/mTORC1, Rictor/mTORC2-YAP/TAZ, and PI3K/Akt/mTOR-HKII maintain fibroblast and pericyte activation and metabolic reprogramming (Jiang et al., 2013; Li et al., 2015; Chen et al., 2023; Humphreys et al., 2010; Gui et al., 2018; Chen et al., 2011). Future strategies should prioritize kidney- or cell-type-selective drug delivery, short-duration interventions, and combination therapies tailored to the fibrotic microenvironment, rather than long-term systemic inhibition of mTORC2 or glycolysis.

Patient stratification and mechanistic validation must also progress from static biomarkers toward multi-layered evidence loops. Biofluid-derived cells, extracellular vesicles, proteomics, and metabolomics offer candidate biomarkers, but these must be mapped against spatial omics, phosphoproteins, redox modifications, and long-term clinical outcomes in renal tissue. For traditional Chinese medicine and natural products, observing changes in p-Akt, Nrf2, LC3, or ROS alone is insufficient to prove mechanism; studies must define target cells and subcellular locations, validate key targets via blocking experiments, and evaluate functional outcomes such as mitochondrial respiration, autophagic flux, lipid peroxidation, and ECM production. The ultimate goal of precision PI3K/Akt-redox therapy is to shorten cell residence time in maladaptive states and disrupt the trans-cellular pathological feedback loops driving CKD progression. A comprehensive summary of current pharmacological interventions targeting the PI3K/Akt-redox axis, along with their candidate targets and clinical translational boundaries in CKD, is presented in Table 1. The cell-, stage-, and compartment-dependent functions underlying these therapeutic considerations are compared in Table 2.

**TABLE 1:** Pharmacological interventions, candidate targets, and translational boundaries related to PI3K/Akt-redox in CKD.

Representative drugs or intervention strategies	Primary targets or signaling branches	Primary target cells/subcellular regions	Applicable disease stages	Main pharmacological effects	Key limitations and potential risks	Evidence types and references
Bardoxolone methyl	Keap1-Nrf2 antioxidant transcriptional network	Multiple renal cell types and systemic tissues	Type 2 diabetes mellitus with CKD	Increased eGFR in the BEAM study; increased inulin clearance-measured GFR in the TSUBAKI study	The BEACON study failed to reduce the composite risk of ESKD or cardiovascular death and increased heart failure events; elevated glomerular filtration rate cannot be directly equated with reversal of structural renal damage	Clinical intervention evidence (Pergola et al., 2011), (de Zeeuw et al., 2013), (Nangaku et al., 2020)
Rapamycin/sirolimus and mTORC1 inhibition	mTORC1	Podocytes, renal fibroblasts, and other renal cell types	Diabetic kidney disease, proteinuric nephropathy, or fibrosis models with mTORC1 hyperactivation	Moderate inhibition of aberrant mTORC1 activity alleviates podocyte hypertrophy, partial proteinuric phenotypes, as well as fibroblast activation and ECM production	Podocytes require a baseline level of mTORC1 activity to maintain the filtration barrier; chronic or excessive inhibition reduces slit diaphragm protein expression, inducing or aggravating proteinuria, and higher sirolimus exposure is associated with new-onset FSGS	Animal genetic, pharmacological, and clinical safety evidence (Inoki et al., 2011; Gödel et al., 2011; Jiang et al., 2013), (Torras et al., 2009; Letavernier et al., 2007; Stallone et al., 2011)
FGF1ΔHBS	PI3K/Akt-GSK3β-Nrf2	Renal parenchymal cells	Experimental CKD	Enhances PI3K/Akt-associated antioxidant responses, suppresses oxidative stress and inflammation, and improves experimental CKD phenotypes	Primarily based on animal experiments; target cell types, subcellular localization, and long-term Akt activation effects remain incompletely clarified	Animal pharmacology evidence (Wang et al., 2019)
Tempol, Salvianolic acid B	PI3K/Akt-Nrf2	Predominantly tubular epithelial cells	Ischemic or contrast-induced AKI	Reduces ROS and oxidative damage, enhances Nrf2-related antioxidant responses, and attenuates acute kidney injury	Evidence derives mainly from acute injury animal models; compounds are not highly selective PI3K/Akt agonists and cannot be directly extrapolated to chronic CKD or long-term pathway activation	Animal pharmacology evidence (Zhang et al., 2016), (Tongqiang et al., 2016)
Gastrin	PI3K/Akt-BAD anti-apoptotic branch	Tubular epithelial cells and mitochondrial apoptotic pathways	Renal ischemia-reperfusion injury	Promotes Akt and Bad phosphorylation, inhibits caspase-dependent apoptosis, and attenuates acute tubular injury	Primarily supports acute-phase protection; whether long-term enhancement of anti-apoptotic signaling prolongs injured cell survival or affects maladaptive repair remains unclear	Animal and cellular pharmacology evidence (Liu et al., 2020a)
Anti-ferroptotic interventions, including salidroside, chicoric acid, and apigenin	PI3K/Akt, PAQR3-p110α, and GPX4/ACSL4-related lipid peroxidation regulation	Predominantly renal tubular epithelial cells	Ischemia-reperfusion, diabetic kidney disease, and hypertensive kidney injury models	Enhances PI3K/Akt signaling in corresponding models, restores GPX4 or reduces ACSL4, ROS, and lipid peroxidation levels, thereby attenuating ferroptosis and renal injury	Mostly natural product studies with limited direct target, selectivity, and pharmacokinetic data; reversal of effects using inhibitors like LY294002 demonstrates pathway involvement but does not prove PI3K/Akt is the sole direct target	Animal and cellular pharmacology evidence (Tang et al., 2023), (Zhang et al., 2024), (Zhang et al., 2025)
RRM2 enhancement strategy	PI3K/Akt-Nrf2-SLC7A11/GSH/GPX4	High-glucose-stimulated HK-2 tubular epithelial cells	Cellular models of diabetic kidney disease	RRM2 overexpression enhances PI3K/Akt/Nrf2 signaling, restores SLC7A11-, GSH-, and GPX4-related antioxidant defenses, and reduces ROS, Fe²⁺, and lipid peroxidation	Cellular overexpression experiments; RRM2 is a candidate molecular target and cannot yet be considered a mature pharmacological intervention	Cellular genetic manipulation evidence (Gao et al., 2025)
4-Phenylbutyric acid (4-PBA)	Endoplasmic reticulum stress and protein folding homeostasis	Podocyte endoplasmic reticulum	Podocyte injury models induced by mTORC1 hyperactivation	Reduces ER stress markers such as GRP78, partially mitigating podocyte phenotypic transition and cell loss	Fails to restore normal membrane localization of nephrin and does not significantly improve proteinuria, suggesting that alleviating ER stress alone is insufficient to repair an intact filtration barrier	Animal mechanistic and pharmacological evidence (Inoki et al., 2011)
Rheb/mTORC1 inhibition strategy	Rheb-mTORC1-S6K/4E-BP1	Activated renal fibroblasts and myofibroblasts	Renal interstitial fibrosis stage	Inhibits TGF-β1-induced fibroblast activation, protein synthesis, and ECM production, attenuating experimental renal fibrosis	Lacks mature fibroblast-selective drug delivery platforms; systemic mTORC1 inhibition may impair podocyte, metabolic, and immune homeostasis	Cellular, animal, and genetic evidence (Jiang et al., 2013)
Rictor/mTORC2-YAP/TAZ inhibition strategy	Rictor/mTORC2-Akt and mTORC2-YAP/TAZ	Renal fibroblasts	Chronic renal fibrosis stage	Reduces expression of α-SMA, fibronectin, and collagen, suppressing TGF-β1-induced fibroblast activation and fibrosis	Highly selective mTORC2 inhibitors for clinical use are currently lacking; mTORC2 is also involved in podocyte stress adaptation, cytoskeletal regulation, and MAM calcium homeostasis, so systemic inhibition may cause off-target damage	Cellular, animal, and genetic evidence (Gödel et al., 2011), (Li et al., 2015), (Betz et al., 2013), (Gui et al., 2018)
PI3K/Akt/mTOR-HKII or glycolysis inhibition strategy	PI3K/Akt/mTOR-HKII metabolic axis	Renal pericytes and pericyte-derived myofibroblast-like cells	Pericyte-to-myofibroblast transition and fibrosis stage	Reduces HKII expression, glucose consumption, and glycolysis, suppressing the transition of pericytes toward myofibroblast-like states	Relies primarily on *in vitro* primary renal pericytes; cell-specific *in vivo* causal validation is lacking, and systemic inhibition of glycolysis may also affect normal repair cells	Primary cell mechanistic evidence (Chen et al., 2023)
AKT1/AKT2 isoform-selective inhibition strategy	AKT1- or AKT2-GSK3β/Snail and β-catenin signaling	Tubular epithelial cells	AKI-to-CKD transition phase and maladaptive repair stage	*Akt1* deficiency attenuates tubular dedifferentiation, apoptosis, and fibrosis; *AKT2* inhibition weakens TGF-β1-induced EMT-like remodeling	Acute-phase mitochondrial AKT1 helps maintain oxidative phosphorylation and cell survival; mature drugs with combined tubular, isoform, and temporal window selectivity are lacking, and premature inhibition may exacerbate acute injury	Animal genetic and cellular mechanistic evidence (Lin et al., 2021), (Kim et al., 2021), (Kattla et al., 2008; Lan et al., 2014; Kim et al., 2023)
Myeloid PI3Kγ inhibition or PTEN functional restoration strategy	PI3Kγ and PTEN-PI3K/Akt	Monocytes and macrophages	Chronic inflammatory recruitment and fibrosis maintenance stage	Theoretically reduces myeloid cell chemotaxis, persistent infiltration, and profibrotic paracrine signaling; myeloid PTEN deficiency aggravates renal inflammation and fibrosis	PI3K/Akt is also involved in macrophage survival, phagocytic clearance, and inflammatory resolution; PI3K/Akt can either promote or limit TLR-induced inflammatory outputs, necessitating distinction of stimuli and cell states	General myeloid mechanisms and renal genetic evidence (Linton et al., 2019; Hirsch et al., 2000; An et al., 2022), (Ojaniemi et al., 2003), (Guha and Mackman, 2002)
Stage-specific autophagy/TFEB modulation strategy	Akt/mTOR-ULK1, TFEB, and autophagy-lysosome system	Proximal tubules and other renal parenchymal cells	Acute kidney injury phase and chronic fibrosis phase	Maintaining autophagic flux in the acute phase helps clear abnormal proteins and damaged mitochondria; in certain chronic models, suppressing persistent aberrant autophagy may reduce tubular cell death, inflammatory mediator release, and fibrosis	Enhanced autophagy initiation does not equate to improved autophagic flux; proteinuria can cause impairment in autophagic processing and lysosomal degradation, whereas persistent autophagy or TFEB-related programs in UUO and adenine CKD models may participate in maladaptive remodeling	Basic mechanisms, animal genetics, and pharmacological evidence (Mizushima and Komatsu, 2011; Sancak et al., 2008; Sancak et al., 2010; Inoki et al., 2002; Menon et al., 2014; Kim et al., 2011; Koyano et al., 2014; Matsuda et al., 2010; Narendra et al., 2008), (Kimura et al., 2011; Nolin et al., 2016; Livingston et al., 2016; Yuan et al., 2021)

**TABLE 2:** Cell-, disease stage-, and compartment-dependent functions of PI3K/Akt-redox signaling in CKD.

Renal cell type	Normal/homeostatic state	Acute stress or adaptive response	Chronic injury or fibrosis	Major organelles or microdomains and key nodes	Translational implication
Podocytes	Balanced insulin-PI3K/Akt, mTORC1/mTORC2, and basal autophagy maintain the slit diaphragm and cytoskeleton ((Inoki et al., 2011), (Gödel et al., 2011), (Welsh et al., 2010), (Hartleben et al., 2010))	AKT2, mTORC2, and basal autophagy support podocyte survival and adaptation to CKD-related or protein-overload stress ((Gödel et al., 2011), (Canaud et al., 2013), (Hartleben et al., 2010))	Sustained mTORC1 activity, endoplasmic reticulum stress, NOX4/mitochondrial ROS, and defective autophagy promote foot-process effacement and proteinuria ((Inoki et al., 2011), (Gödel et al., 2011), (Eid et al., 2013), (Hartleben et al., 2010; Lindenmeyer et al., 2008; Vollenbröker et al., 2009; Wang et al., 2021; Ducasa et al., 2019))	Slit diaphragm, cytoskeleton, endoplasmic reticulum, mitochondria, and lysosomes; AKT2, mTORC1/2, NOX4	Preserve the physiological mTOR-autophagy window; consider podocyte-directed rather than systemic inhibition
Tubular epithelium	Fatty acid oxidation, oxidative phosphorylation, and basal autophagy maintain transport and energy homeostasis ((Kang et al., 2015), (Kimura et al., 2011))	Mitochondrial AKT1, Nrf2, TEAD1-PGC-1α, and autophagic/mitochondrial quality control support survival (14- (Lin et al., 2021), (Martin et al., 2004), (Tran et al., 2025), (Kimura et al., 2011))	KT1/AKT2-GSK3β-Snail, SOX4-Smad3, Akt/mTOR, and autophagy imbalance promote tubular dedifferentiation and profibrotic repair, whereas ferroptosis contributes to tubular injury ((Huang et al., 2016), (Kim et al., 2021), (Tang et al., 2023; Zhang et al., 2024; Gao et al., 2025; Zhang et al., 2025; Kimura et al., 2011), (Kattla et al., 2008; Lan et al., 2014; Kim et al., 2023; Du et al., 2026; Kimura et al., 2011; Nolin et al., 2016; Livingston et al., 2016; Yuan et al., 2021))	Mitochondria, endoplasmic reticulum, autolysosomes, and lipid membranes; PTEN/PIP3, Nrf2, mTOR, GPX4/SLC7A11	Use disease-stage-specific tubular and mitochondrial delivery; avoid suppressing early protective signaling
Endothelium	Akt-eNOS-NO signaling maintains perfusion, barrier function, and vascular quiescence ((Dimmeler et al., 1999; Fulton et al., 1999; Förstermann and Sessa, 2012))	Preservation of endothelial survival and Akt-eNOS signaling may support microvascular integrity and tissue perfusion during acute injury ((Yang et al., 2018), (Dimmeler et al., 1999), (Fulton et al., 1999))	NOX- and mitochondrial ROS-NF-κB signaling promotes endothelial activation, capillary rarefaction, and hypoxia ((Kida et al., 2014; Yang et al., 2018; Prommer et al., 2018), (Du et al., 2001; Tumur et al., 2010; D'Apolito et al., 2015; Abid et al., 2007))	Plasma membrane and mitochondria; AKT, eNOS, NOX, NF-κB	Preserve Akt-eNOS-NO while selectively limiting inflammatory ROS sources
Macrophages/myeloid cells	Tissue surveillance, efferocytosis, and immune homeostasis predominate ((Liu et al., 2020b; Conway et al., 2020; Linton et al., 2019))	PI3Kγ-dependent recruitment and Akt-dependent survival or efferocytosis shape early injury responses; CSF-1-dependent signaling can support repair ((Linton et al., 2019), (Hirsch et al., 2000), (Furuichi et al., 2006), (Menke et al., 2009))	Reduced PTEN restraint, STAT6 and JMJD3-IRF4 programs, cGAS-STING, and NLRP3 promote persistent inflammation and profibrotic crosstalk ((Jiao et al., 2025), (An et al., 2022; Jiao et al., 2021a; Jiao et al., 2021b; An et al., 2023; Liang et al., 2022), (Zhou et al., 2011; Nakahira et al., 2011; Li et al., 2025), (Peng et al., 2023), (Cohen et al., 2024))	Cytosol, mitochondria, and phagolysosomes; PI3Kγ, PTEN, Nrf2, cGAS-STING, NLRP3	Target defined profibrotic states while preserving efferocytosis and inflammatory resolution
Fibroblasts/pericytes	Quiescent fibroblasts and pericytes occupy interstitial and perivascular niches in the healthy kidney ((Lake et al., 2023), (Abedini et al., 2024), (Humphreys et al., 2010))	Following kidney injury, pericyte detachment and proliferation initiate the transition toward myofibroblast-like states ((Humphreys et al., 2010), (Chen et al., 2011))	Rheb-mTORC1, Rictor-mTORC2-Akt-YAP/TAZ-TEAD, and PI3K/Akt/mTOR-HKII sustain myofibroblast activation and ECM production ((Abedini et al., 2024; Jiang et al., 2013; Li et al., 2015; Chen et al., 2023), (Gui et al., 2018), (Chen et al., 2011))	Growth-factor receptor domains, lysosomal mTORC1, cytoskeleton, and metabolic compartments; mTORC1/2, YAP/TAZ-TEAD, HKII.	Favor fibroblast- or fibrotic-niche-selective, time-limited delivery over chronic systemic mTOR blockade

### 4.4 Kidney cell- and organelle-targeted drug delivery

Because PI3K/Akt-redox signaling is highly context-dependent, drug delivery systems (DDSs) represent an important component of precision therapy rather than merely a formulation consideration. A clinically oriented DDS should account for the target cell and disease stage, administration route, carrier size and charge, targeting ligand, release mechanism, and pharmacodynamic markers of target engagement.

Proximal tubular epithelial cells are particularly accessible because filtered low-molecular-weight ligands can exploit megalin/cubilin-mediated endocytosis. Renal tubule-targeting carbohydrate (RENTAC) conjugates have delivered antisense peptide nucleic acids to proximal tubules and attenuated renal fibrosis in mice (Kumar et al., 2024). Polymeric mesoscale nanoparticles have likewise shown preferential renal tubular accumulation following systemic administration (Williams et al., 2018).

Podocyte-directed delivery is more challenging because access is constrained by the glomerular filtration barrier and may vary with proteinuria and structural injury. In experimental diabetic nephropathy, a biomimetic nanoplatform co-delivered an mTORC1 inhibitor and a ROS-scavenging agent to glomerular and podocyte regions, reducing proteinuria and glomerulosclerosis (Fan et al., 2023). Transient receptor potential cation channel subfamily C member 6 (TRPC6)-targeted nanobubbles have also enhanced podocyte-directed dexamethasone delivery together with ultrasound-triggered drug release and imaging in adriamycin nephropathy (Wu L. et al., 2025). However, these approaches remain preclinical and require further validation across different podocytopathies.

At the organelle level, the mitochondria-targeted Szeto-Schiller peptide 31 (SS-31) reduced mitochondrial oxidative injury in experimental diabetic kidney disease (Hou et al., 2016). A folic acid-decorated hierarchical nanodrug also achieved sequential kidney, injured tubular cell, and mitochondrial/nuclear targeting in acute kidney injury (Chen et al., 2025). In principle, these platforms could preserve mitochondrial integrity during acute injury while preferentially delivering mTOR, NADPH oxidase, or ferroptosis inhibitors to maladaptive cells during chronic repair.

Clinical translation will require quantitative biodistribution studies in human-relevant models, evaluation of target-receptor variability and off-target uptake, long-term clearance and immunogenicity testing, reproducible manufacturing, and biomarker-based confirmation of target engagement.

## 5 Conclusion

The relationship between PI3K/Akt signaling and oxidative stress in CKD cannot be summarized simply as antioxidant protection or pro-fibrotic action. Reversible PTEN oxidation, localized PIP3/Akt activation, GSK3β/Nrf2 antioxidant regulation, the podocyte mTOR homeostatic window, AKT1 involvement in AKI-to-CKD transition, mTORC1/2-mediated interstitial cell activation, and tubular ferroptosis collectively establish PI3K/Akt as a context-dependent redox regulatory hub.

Future PI3K/Akt-redox research in CKD should move beyond tissue-level pathway averages toward cell-state- and spatially resolved analyses. The source, duration, and localization of ROS shape signal inputs; the oxidation state of PTEN and other phosphatases tunes the Akt activation threshold; organellar microdomains influence ROS generation and cell death sensitivity; downstream Nrf2, FOXO, NF-κB, mTOR, autophagy, and ferroptosis branches dictate signal outputs; and cellular states alongside their spatial neighborhoods determine final pathological outcomes. This framework helps explain the coexistence of protective and pathogenic PI3K/Akt effects in CKD, providing an analytical basis for precision interventions that integrate single-cell and spatial omics, phosphoproteomics, redox proteomics, and metabolomics.

## Glossary

**4-HNE**: 4-Hydroxynonenal
**4-PBA**: 4-Phenylbutyric acid
**4E-BP1**: Eukaryotic translation initiation factor 4 E-binding protein 1
**ABCA1**: ATP-binding cassette transporter A1
**ACSL4**: Acyl-CoA synthetase long-chain family member 4
**ACTA2**: Actin alpha 2, smooth muscle
**AGE/AGEs**: Advanced glycation end product(s)
**AIM2**: Absent in melanoma 2
**AKI**: Acute kidney injury
**AKI-to-CKD**: Acute kidney injury-to-chronic kidney disease transition
**Akt/PKB**: Protein kinase B
**AKT1**: AKT serine/threonine kinase 1
**Akt1**: AKT serine/threonine kinase 1 gene
**AKT2**: AKT serine/threonine kinase 2
**Akt2**: AKT serine/threonine kinase 2 gene
**AKT3**: AKT serine/threonine kinase 3
**AMPK**: AMP-activated protein kinase
**ANKRD1**: Ankyrin repeat domain 1
**ARE**: Antioxidant response element
**Atg5**: Autophagy-related 5 gene
**Atg7**: Autophagy-related 7 gene
**ATP**: Adenosine triphosphate
**AYAME**: A Phase 3 Study of Bardoxolone Methyl in Patients with Diabetic Kidney Disease
**BAD**: BCL2-associated agonist of cell death
**BEACON**: Bardoxolone Methyl Evaluation in Patients with Chronic Kidney Disease and Type 2 Diabetes Mellitus: the Occurrence of Renal Events
**BEAM**: 52-Week Bardoxolone Methyl Treatment: Renal Function in CKD/Type 2 Diabetes
**Ca** ^**2**+^: Calcium ion
**CCL2**: C-C motif chemokine ligand 2
**CD2AP**: CD2-associated protein
**Cdk5**: Cyclin-dependent kinase 5
**cGAMP**: Cyclic guanosine monophosphate-adenosine monophosphate
**cGAS**: Cyclic GMP-AMP synthase
**CKD**: Chronic kidney disease
**COL1A1**: Collagen type I alpha 1 chain
**CSF-1**: Colony-stimulating factor 1
**CSF-1R**: Colony-stimulating factor 1 receptor
**CTGF**: Connective tissue growth factor
**DAMPs**: Damage-associated molecular patterns
**DDS/DDSs**: Drug delivery system(s)
**DKD**: Diabetic kidney disease
**dsDNA**: Double-stranded DNA
**ECM**: Extracellular matrix
**eGFR**: Estimated glomerular filtration rate
**EMT**: Epithelial-mesenchymal transition
**eNOS**: Endothelial nitric oxide synthase
**ER**: Endoplasmic reticulum
**ERK1/2**: Extracellular signal-regulated kinase 1/2
**ERO1**: Endoplasmic reticulum oxidoreductin 1
**ESKD**: End-stage kidney disease
**ETC**: Electron transport chain
**FAM3A**: Family with sequence similarity 3 member A
**Fe** ^**2**+^: Ferrous ion
**FGF1ΔHBS**: Heparin-binding-site-deficient fibroblast growth factor 1
**FN1**: Fibronectin 1
**FOLR2**: Folate receptor beta
**Foxd1**: Forkhead box D1 gene
**FOXO**: Forkhead box O
**FSGS**: Focal segmental glomerulosclerosis
**GAPDH**: Glyceraldehyde-3-phosphate dehydrogenase
**GBM**: Glomerular basement membrane
**GCLC**: Glutamate-cysteine ligase catalytic subunit
**GCLM**: Glutamate-cysteine ligase modifier subunit
**GFR**: Glomerular filtration rate
**GPX4**: Glutathione peroxidase 4
**GRP78**: 78-kDa glucose-regulated protein
**GSDMD**: Gasdermin D
**GSH**: Reduced glutathione
**GSK3β**: Glycogen synthase kinase 3 beta
**GSSG**: Glutathione disulfide
**HIF-1α**: Hypoxia-inducible factor 1 alpha
**HK-2**: Human kidney-2 cell line
**HKII**: Hexokinase II
**HO-1**: Heme oxygenase 1
**H**_**2**_ **O**_**2**_: Hydrogen peroxide
**ICAM-1**: Intercellular adhesion molecule 1
**IFN**: Interferon
**IL-13**: Interleukin 13
**IL-18**: Interleukin 18
**IL-1β**: Interleukin 1 beta
**IL-4**: Interleukin 4
**IL-6**: Interleukin 6
**IL1B**: Interleukin 1 beta gene
**IL6**: Interleukin 6 gene
**IP3R3**: Inositol 1,4,5-trisphosphate receptor type 3
**IRF3**: Interferon regulatory factor 3
**IRF4**: Interferon regulatory factor 4
**JMJD3**: Jumonji domain-containing protein 3
**KCa3.1**: Intermediate-conductance calcium-activated potassium channel KCa3.1
**KDM6B**: Lysine demethylase 6 B (also known as JMJD3)
**Keap1**: Kelch-like ECH-associated protein 1
**LC3-II**: Microtubule-associated protein 1 light chain 3, lipidated form II
**LPCAT3**: Lysophosphatidylcholine acyltransferase 3
**M1/M2**: Classically activated/alternatively activated macrophage nomenclature
**MAM/MAMs**: Mitochondria-associated endoplasmic reticulum membrane(s)
**MAPK**: Mitogen-activated protein kinase
**MCP-1**: Monocyte chemoattractant protein 1
**MIP-1α**: Macrophage inflammatory protein 1 alpha
**mtDNA**: Mitochondrial DNA
**mTOR**: Mechanistic target of rapamycin
**mTORC1**: Mechanistic target of rapamycin complex 1
**mTORC2**: Mechanistic target of rapamycin complex 2
**mtROS**: Mitochondrial reactive oxygen species
**NADPH**: Reduced nicotinamide adenine dinucleotide phosphate
**Neh2**: Nrf2-ECH homology 2 domain
**NEK7**: NIMA-related kinase 7
**NF-κB**: Nuclear factor kappa B
**NLRP3**: NLR family pyrin domain-containing 3
NO: Nitric oxide
**NOX**: NADPH oxidase
**NOX2**: NADPH oxidase 2
**NOX4**: NADPH oxidase 4
**NQO1**: NAD(P)H quinone dehydrogenase 1
**Nrf2**: Nuclear factor erythroid 2-related factor 2
**NRK-52E**: Normal rat kidney epithelial cell line 52 E
**O-GlcNAc**: O-linked beta-N-acetylglucosamine
**OXPHOS**: Oxidative phosphorylation
**O**_**2**_: Molecular oxygen
**p-Akt**: Phosphorylated Akt
**p-eNOS**: Phosphorylated endothelial nitric oxide synthase
**p-FOXO**: Phosphorylated Forkhead box O
**p-GSK3β**: Phosphorylated glycogen synthase kinase 3 beta
**p-mTOR**: Phosphorylated mechanistic target of rapamycin
**p-p65**: Phosphorylated NF-κB p65 subunit
**p-S6**: Phosphorylated ribosomal protein S6
**p110α**: Phosphoinositide 3-kinase catalytic subunit p110 alpha
**P2Y1**: P2Y purinergic receptor 1
**p38 MAPK**: p38 mitogen-activated protein kinase
**p62/SQSTM1**: Sequestosome 1
**PAQR3**: Progestin and adipoQ receptor family member 3
**PDGF**: Platelet-derived growth factor
**PDGFR**: Platelet-derived growth factor receptor
**PDGFRβ**: Platelet-derived growth factor receptor beta
**PDI**: Protein disulfide isomerase
**PERK**: Protein kinase R-like endoplasmic reticulum kinase
**PGC-1α**: Peroxisome proliferator-activated receptor gamma coactivator 1-alpha
**PI3K**: Phosphoinositide 3-kinase
**PI3Kγ**: Phosphoinositide 3-kinase gamma
**PINK1**: PTEN-induced kinase 1
**PIP2**: Phosphatidylinositol 4,5-bisphosphate
**PIP3**: Phosphatidylinositol 3,4,5-trisphosphate
**PKC**: Protein kinase C
**PL-OH**: Phospholipid alcohol
**PL-OOH**: Phospholipid hydroperoxide
**PRDX4**: Peroxiredoxin 4
**PTEN**: Phosphatase and tensin homolog
**PTP1B**: Protein tyrosine phosphatase 1 B
**PTPs**: Protein tyrosine phosphatases
**PUFA**: Polyunsaturated fatty acid
**PUFA-PLs**: Polyunsaturated fatty acid-containing phospholipids
**Rag GTPases**: Ras-related GTP-binding proteins
**Raptor**: Regulatory-associated protein of mTOR
**RENTAC**: Renal tubule-targeting carbohydrate
**Rheb**: Ras homolog enriched in brain
**Rictor/RICTOR**: Rapamycin-insensitive companion of mTOR (protein); RPTOR-independent companion of MTOR complex 2 (gene)
**ROS**: Reactive oxygen species
**Rptor**: Regulatory-associated protein of MTOR complex 1 gene
**RRM2**: Ribonucleotide reductase regulatory subunit M2
**RTK**: Receptor tyrosine kinase
**S6K**: Ribosomal protein S6 kinase
**scRNA-seq**: Single-cell RNA sequencing
**SHP**2: Src homology 2 domain-containing protein tyrosine phosphatase 2
**Sirt1**: Sirtuin 1
**SLC7A11**: Solute carrier family 7 member 11
**SMAD3/Smad3**: SMAD family member 3
**sMaf**: Small musculoaponeurotic fibrosarcoma protein
**snRNA-seq**: Single-nucleus RNA sequencing
**SOX4**: SRY-box transcription factor 4
**Src**: Proto-oncogene tyrosine-protein kinase Src
**SREBP**: Sterol regulatory element-binding protein
**SS-31**: Szeto-Schiller peptide 31
**STAT6**: Signal transducer and activator of transcription 6
**STING**: Stimulator of interferon genes
**TAZ**: Transcriptional coactivator with PDZ-binding motif
**TBK1**: TANK-binding kinase 1
**TEAD/TEAD1-4**: TEA domain transcription factor(s)/TEA domain family members 1-4
**TFEB**: Transcription factor EB
**TGF-β**: Transforming growth factor beta
**TGF-β1**: Transforming growth factor beta 1
**TGFBR2**: Transforming growth factor beta receptor 2
**Tgfbr2**: Transforming growth factor beta receptor 2 gene
**TLR4**: Toll-like receptor 4
**TMAO**: Trimethylamine N-oxide
**TRPC6**: Transient receptor potential cation channel subfamily C member 6
**TSC**: Tuberous sclerosis complex
**TSC1**: Tuberous sclerosis complex 1
**Tsc1**: Tuberous sclerosis complex 1 gene
**TSC2**: Tuberous sclerosis complex 2
**TSUBAKI**: Phase 2 Study of Bardoxolone Methyl in Patients with Chronic Kidney Disease and Type 2 Diabetes
**ULK1**: Unc-51-like autophagy-activating kinase 1
**UPR**: Unfolded protein response
**UUO**: Unilateral ureteral obstruction
**VCAM**-1: Vascular cell adhesion molecule 1
**VEGF**: Vascular endothelial growth factor
**WNT**: Wingless-related integration site signaling pathway
**YAP**: Yes-associated protein
**TAZ**: Transcriptional coactivator with PDZ-binding motif
**α-SMA**: Alpha-smooth muscle actin
